# Genome-wide identification of MST, SUT and SWEET family sugar transporters in root parasitic angiosperms and analysis of their expression during host parasitism

**DOI:** 10.1186/s12870-019-1786-y

**Published:** 2019-05-14

**Authors:** Vikram A. Misra, Eric K. Wafula, Yu Wang, Claude W. dePamphilis, Michael P. Timko

**Affiliations:** 1 Department of Biology, University of Virginia, Gilmer Hall 044, Charlottesville, VA 22904 USA; 2 Department of Biology, Penn State University, University Park, PA 16802 USA; 3 Present Address: Center for Quantitative Sciences, Vanderbilt University, 2220 Pierce Avenue, 571 Preston Research Building, Nashville, TN 37232-6848 USA

**Keywords:** Parasitic plant, Sugar transporter, MST, SUT, SWEET, Expression, *Orobanchaceae*

## Abstract

**Background:**

Root parasitic weeds are a major constraint to crop production worldwide causing significant yearly losses in yield and economic value. These parasites cause their destruction by attaching to their hosts with a unique organ, the haustorium, that allows them to obtain the nutrients (sugars, amino acids, etc.) needed to complete their lifecycle. Parasitic weeds differ in their nutritional requirements and degree of host dependency and the differential expression of sugar transporters is likely to be a critical component in the parasite’s post-attachment survival.

**Results:**

We identified gene families encoding monosaccharide transporters (MSTs), sucrose transporters (SUTs), and SWEETs (Sugars Will Eventually be Exported Transporters) in three root-parasitic weeds differing in host dependency: *Triphysaria versicolor* (facultative hemiparasite), *Phelipanche aegyptiaca* (holoparasite), and *Striga hermonthica* (obligate hemiparasite). The phylogenetic relationship and differential expression profiles of these genes throughout parasite development were examined to uncover differences existing among parasites with different levels of host dependence. Differences in estimated gene numbers are found among the three parasites, and orthologs within the different sugar transporter gene families are found to be either conserved among the parasites in their expression profiles throughout development, or to display parasite-specific differences in developmentally-timed expression. For example, MST genes in the pGLT clade express most highly before host connection in *Striga* and *Triphysaria* but not *Phelipanche*, whereas genes in the MST ERD6-like clade are highly expressed in the post-connection growth stages of *Phelipanche* but highest in the germination and reproduction stages in *Striga.* Whether such differences reflect changes resulting from differential host dependence levels is not known.

**Conclusions:**

While it is tempting to speculate that differences in estimated gene numbers and expression profiles among members of MST, SUT and SWEET gene families in *Phelipanche*, *Striga* and *Triphysaria* reflect the parasites’ levels of host dependence, additional evidence that altered transporter gene expression is causative versus consequential is needed. Our findings identify potential targets for directed manipulation that will allow for a better understanding of the nutrient transport process and perhaps a means for controlling the devastating effects of these parasites on crop productivity.

**Electronic supplementary material:**

The online version of this article (10.1186/s12870-019-1786-y) contains supplementary material, which is available to authorized users.

## Introduction

Root-parasitic weeds in the family *Orobanchaceae* are among the major constraints to crop production worldwide [1–3], causing losses in crop yield and economic value estimated to be in excess of several billion USD annually [2, 3]. Among the hosts to these parasites are many of the world’s most important food and forage grasses (corn, sorghum, millet and rice) and grain legumes (cowpea, peanut) with yield losses ranging from minimal to 100% in areas throughout their range [4–6]. Currently, measures limiting the effects and spread of these parasites are not effective. Compounding these control difficulties is the fact that the seeds of the various *Orobanchaceae* can remain viable in the soil for many years [7] making eradication difficult.

It is now well documented that progression through the parasite life-cycle is dependent upon a series of host-derived chemical cues that lead to critical developmental changes throughout the host-parasite interaction. For example, parasite seed germination only occurs after a period of after-ripening and preconditioning in the soil, and in response to the presence of host-derived chemical stimulants (e.g., strigolactones) in the rhizosphere [8, 9]. Following germination, the elongating parasite radicle then transforms into a haustorium, the unique organ for host attachment, in response to a second set of host-derived chemical cues referred to as haustorial initiation factors (HIFs) [10, 11]. Once formed, the haustorium then attaches to the host root, develops invasive tissues that transverse the host root cortex, and eventually establish connection with the host vascular system. Coincident with this is the development of specialized cells and tissues that allow the parasite to obtain and utilize host derived nutrients [10]. Most of the damage to the host plant occurs during the early pre-emergent stages of parasite growth as evidenced by a decline in host growth and vigor [1]. With the emergence of the parasite shoot from the soil, it grows to reproductive maturity, flowers and sets seed, thereby completing its life-cycle [9, 10].

Despite their enormous threat to many crops, little research was done until recently on the molecular aspects of host-parasite weed interactions relative to other well-studied disease-causing agents. Most early molecular studies of parasitic plants of the *Orobanchaceae* family focused on plastid genome evolution [12, 13], diversity analysis [14–18], and phylogenetic relationships [19–21] among family members. Subsequently, reports appeared in which investigators attempted to understand the gene expression changes in both hosts and parasites in response to attack by the parasite using both differential gene expression profiling using cDNA library analysis and sequencing of expressed sequence tags (ESTs) from both host and parasite [22–26].

Comparative transcriptomics of three parasitic species in the *Orobanchaceae* (i.e., *Triphysaria versicolor*, *Phelipanche aegyptiaca*, and *Striga hermonthica*) and several related non-parasites was carried out in order to examine the global changes that occurred during the evolution of parasitism in this group and to potentially identify changes required for the parasitic lifestyle [27–29]. Over 3 billion sequence reads from more than 30 tissue-, developmental stage-specific-, and normalized whole plant- libraries were generated and are available for analysis through the Parasitic Plant Genome Project (PPGP; http://ppgp.huck.psu.edu/). A first approach at analyzing these datasets conducted by Wickett et al. [30] showed that while the non-photosynthetic parasitic plant *P. aegyptiaca* had no detectable expression of genes involved in the formation of the photosystems and light harvesting proteins, the genes for chlorophyll biosynthesis were retained, transcribed, and subject to purifying selection, suggesting a function for chlorophyll independent of photosynthetic activity. Subsequently, Yang et al. [31] analyzed the transcriptomic data from *P. aegyptiaca*, *S. hermonthica*, and *T. versicolor* for differential expression and gene expression clustering in order to identify candidate parasitism genes with roles in facilitating parasitic interactions with host plants. These investigators showed that expression shifts, with or without gene duplication, was a common mechanism by which most genes involved with parasitism evolved and they suggested that either adaptive evolution or relaxed selective constraints may have been important in the evolution of haustorial genes. Based on expression data, Yang et al. [31] further suggested that the co-option of genes involved with root and floral development played a key role in the evolution of the haustorium. Using transcriptomic data generated from host-parasite interface tissue samples prepared by laser capture dissection, Honaas et al. [32] showed that parasitic plant gene expression patterns change in response to the nature of the host being attacked. Moreover, among the genes differentially expressed at the interface are various sugar transporters, indicating a likely crucial role during the host-parasite interaction.

Sugar transporters are broadly characterized into three major types: monosaccharide transporters (MSTs), sucrose transporters (SUTs), and SWEETs (Sugars Will Eventually be Exported Transporters). MSTs and SUTs function in sugar influx [33], whereas the SWEETs (also known as *Medicago truncatula* nodulin 3 (MtN3)/saliva [34]) play important roles in phloem transport [35] where they primarily function in sucrose efflux and bidirectional sugar transport [35–37].

MSTs are Sugar_tr domain (PF00083) containing members of the major facilitator superfamily (MFS) class of transporters. Structurally, MFS transporters usually contain 12 transmembrane (TM) domains [37–39]. Seven MST clades have been defined in plants: (i) the Early Response to Dehydration 6-like (ERD6-like) clade [40] (sometimes abbreviated as ESL); (ii) the plastidic glucose translocator (pGLT) clade, which contains the SGB1 (suppressor of G protein beta 1) sub-clade [37]; (iii) the inositol transporters (INT) and (iv) the tonoplastic monosaccharide transporters (TMT) clades [41], both of whose members localize to the tonoplast and plasma membrane [41–43] and are involved in vacuolar monosaccharide transport [37, 44]; (v) the polyol/monosaccharide transporter (PMT) clade, which is referred to as PLT in Lalonde & Frommer [41]; (vi) the vacuolar glucose transporters (VGT) clade; and (vii) the sugar transport protein (STP) clade [38, 41], whose members play a variety of roles, including interaction with symbiotic and pathogenic fungi [37], pollen development, and root development [45].

SUTs, like MSTs, are Sugar_tr domain (PF00083) members of the MFS superfamily [38], but their organization is less clear. The SUTs were originally split into five clades encompassed by SUT1-SUT5 [41, 46]. SUT1 is a dicot specific clade whose members are expressed in companion cells and sieve elements [37] and is proposed to move from companion cells to sieve elements via the endoplasmic reticulum (ER) [46, 47]. SUT1 proteins play a role in phloem loading [48, 49] and unloading [50] and in cellular interactions with symbiotic and pathogenic fungi (reviewed in [37]). Members of the SUT2 clade are found in both monocots and dicots [46] and are expressed in sink cells and, to a lesser extent, source leaves [51]. Like SUT1 clade members, SUT2 clade members can contribute to phloem loading and the transport of sucrose into various sink cells [52]. The SUT4 clade is also expressed in both monocots and dicots [46] and is localized in expression to sieve elements [53] and in source leaves [54]. They are involved in several processes including responses to dehydration and photosynthesis [54] and circadian rhythms [55], to nodule development [56]. SUT gene expression has been shown to change under different physiological conditions associated with source-to-sink sucrose transport [57]. Peng et al. [58] split the SUTs into two subfamilies: Ancient Group 1 (AG1) and Ancient Group 2 (AG2). Within AG1 are two clades, the “Type I” clade unique to dicots and the “Type III” found in both monocots and dicots. There are also two clades within AG2, the “Type II” found in both monocots and dicots and “Type IIB” which is monocot specific. According to Peng et al. [58], the SUT1 group reported by Kuhn & Grof [46] and the SUT4 group reported by Lalonde & Frommer [41] belong to the Type I and Type III clades, respectively. The Type II clade of Peng et al. [58] contains SUT2 from Lalonde & Frommer [41] and the Type IIB group contains SUT3 and SUT5 from Kuhn & Grof [46].

SWEETs are sugar transporters, which like SUTs and MSTs, undergo changes in gene expression under different physiological conditions, changes that are associated with the regulation of source-to-sink sucrose transport [57]. In contrast to MSTs and SUTs, the SWEETs are members of a superfamily of transporters containing seven TM domains [36, 37, 59]. SWEETs are characterized by an MtN3/saliva domain [60] and contain a pair of repeats that span two TM helices, connected by a loop [61]. There are four groups of SWEETs exclusive to plants, numbered I through IV.

Prior studies have established a crucial role for sugar transport in basic plant growth and development, in root and shoot elongation, growth at the meristems, and interactions with commensal and disease-causing microbes [33, 37, 45, 62]. Members of both the MSTs and SUTs have been implicated in controlling the interaction between host plants and pathogenic fungi [37, 63], and SWEETs have been shown to be exploited by pathogens such as *Xanthomonas oryzae* [64]. Apart from an analysis of sucrose transporters in *Phelipanche ramosa* [65] little is currently known about the structure and organization of sugar transporter gene families in parasitic weeds and the role they may play in mediating host-parasite interactions.

It is well documented that parasitic plants differ in their degree of host dependency ranging from facultative hemiparasitic forms (e.g., *Tryphysaria* spp.) which can photosynthesize and complete their lifecycle without host contact, to fully non-photosynthetic holoparasites, including species of *Phelipanche* (syn. *Orobanche*) genus*,* which are totally dependent upon their host. Others, like the obligate hemiparasite *Striga,* are capable of photosynthesis but are host dependent at their early growth stages and remain so later in development for as yet unclear reasons [27–29]. In light of these differences and the importance of nutrient exchange from host to parasite throughout their lifecycle, we hypothesized that differences in the developmental timing and level of expression of sugar transporters may exist among parasites with differing host dependence and that these differences may be important to parasite survival. To address this question, we have characterized the sugar transporter repertoires of three parasitic plants, *Phelipanche aegyptiaca*, *Striga hermonthica*, and *Triphysaria versicolor* that differ in their degree of host dependency and examined the patterns of expression of the various family members in parasite seedlings before and following host attachment and in the early and post-emergent/reproductive stages of parasite growth. In addition, we use analyses of orthogroups to determine whether any variations in expression within a clade can be explained by the possible presence of multiple orthogroups in the same sugar transporter clade. We identify members of the MST, SUT, and SWEET gene families with both general and developmental stage specific expression and speculate on the potential roles of these genes/gene families in controlling host interaction.

## Results

We have identified members of the MST, SUT and SWEET sugar transporter gene families in three parasitic plants, the facultative hemiparasite *Triphysaria versicolor*, the holoparasitic *Phelipanche aegyptiaca*, and the obligate hemiparasite *Striga hermonthica*, and the non-parasite *Mimulus guttatus (*syn. *Erythranthe guttata)* a member of the traditional Scrophulariaceae (now Phrymaceae) [66]*,* using a novel gene discovery pipeline. This discovery pipeline (see Materials and Methods) uses a strict set of rules based upon domain structure to define gene family membership. To identify members of the SUT families and to accommodate the previous phylogenetic organizations of Kuhn & Grof [46] and Lalonde & Frommer [41]. In the present study, we used representative sequences from the rice SUT3 and SUT5 clades and the Arabidopsis SUT1, SUT2, and SUT4 clades to query the parasite datasets. Clades SUT1 – SUT5 are given in the figures presented, and for consistency with prior studies of Peng et al. [58] we treat the SUT1 clade as being synonymous with Type I clade, SUT2 as being synonymous with Type II clade, SUT4 as being synonymous with Type III, and SUT3 and SUT5 clades as being synonymous with Type IIB. As search queries to find parasitic plant SWEET sequences we employed the conserved SWEET domains of 33 rice SWEET gene sequences (21 from the *Japonica* sub-species and 12 from the *Indica* sub-species) and 17 sequences from *A. thaliana*. Table 1 shows the estimated sizes of the MST, SUT and SWEET gene families based on the three parasitic plants transcriptomes studied and the *M. guttatus* genome. These are representative genes in which multiple isoforms are excluded. The obligate hemiparasite *S. hermonthica* has the largest number of expressed MST representative genes, just slightly greater than that found in the facultative parasite *T. versicolor*. This is almost double of that found in the holoparasite *P. aegyptiaca*. *S. hermonthica* also has the largest number of expressed SUTs and SWEETs. The *M. guttatus* genome contains 46 representative MST genes, 4 SUT genes, and 29 representative SWEET genes (Table 1). By comparison, there are 53 MSTs [45] and 9 SUTs [58] in the *Arabidopsis thaliana* genome, while the rice genome has 65 MSTs [67] and 5 SUTs [58]. Yuan & Wang [68] reported that there are 21 SWEETs in the rice genome and 17 in the *A. thaliana* genome. Two points are worth noting here. First, due to the fragmentary nature of transcriptome de novo assemblies, our copy numbers for the parasite species refer to estimated numbers since accurate copy numbers can only be determined when complete genome assemblies and mature annotations become available. Second, the sequence set at the nucleotide and amino acid level was examined for possible isoforms using the following criteria: sequences with attributes such as high similarity to other sequences, similar domain structure, and short (< 0.01) distances on a neighbor joining phylogram. These criteria were used to remove possible isoforms. However, the expression values from isoform-rich and single-isoform filtered datasets were studied.

**Table 1:** Estimated numbers of sugar transporters in three parasitic plant transcriptomes and the *Mimulus* genome. For each group of sugar transporters, the percentage of sequences is given for each taxon on the phylogenetic trees in Figs. 1, 2, 3, 4 and 5 and Additional files 1, 2, 3, 4 and 5. Please note that the percentages calculated in this table are the percentages of only the sequences found in the phylogenetic trees. In the table, “outliers” refer to sequences that were eliminated during the part of the curation phase in which preliminary neighbor-joining trees were made to detect sequences that were on branches longer than 0.3 when related branches had length of under 0.1; these outlier sequences were on branches longer than 0.3

	*Phelipanche aegyptiaca*		*Striga hermonthica*		*Triphysaria versicolor*		*Mimulus guttatus*	
	#	% of Total on Tree	#	% of Total on Tree	#	% of Total on Tree	#	% of Total on Tree
ERD6	5	14.71%	8	12.12%	6	9.52%	6	13.04%
pGLT_SGB	2	5.88%	2	3.03%	3	4.76%	4	8.70%
INT	3	8.82%	7	10.61%	3	4.76%	6	13.04%
TMT	6	17.65%	2	3.03%	3	4.76%	4	8.70%
PMT	7	20.59%	20	30.30%	23	36.51%	7	15.22%
VGT	2	5.88%	2	3.03%	3	4.76%	2	4.35%
STP	9	26.47%	25	37.88%	22	34.92%	17	36.96%
Total MST on Tree	34		66		63		46	
MST Isoforms and Outliers	80		162		174		23	
SUT1	2	50.00%	5	55.56%	3	60.00%	2	50.00%
SUT2	1	25.00%	1	11.11%	1	20.00%	1	25.00%
SUT4	1	25.00%	3	33.33%	1	20.00%	1	25.00%
Total SUT on Tree	4		9		5		4	
SUT Isoforms and Outliers	7		11		10		0	
SWEET Clade I	8	57.14%	8	32.00%	5	38.46%	10	34.48%
SWEET Clade II	0	0.00%	5	20.00%	3	23.08%	3	10.34%
SWEET Clade III	6	42.86%	10	40.00%	5	38.46%	14	48.28%
SWEET Clade IV	0	0.00%	2	8.00%	0	0.00%	2	6.90%
Total SWEET on Tree	14		25		13		29	
Other SWEET Isoforms and Outliers	3		6		1		5	

### Phylogenetic analysis of sugar transporter gene families

**Fig. 1:**
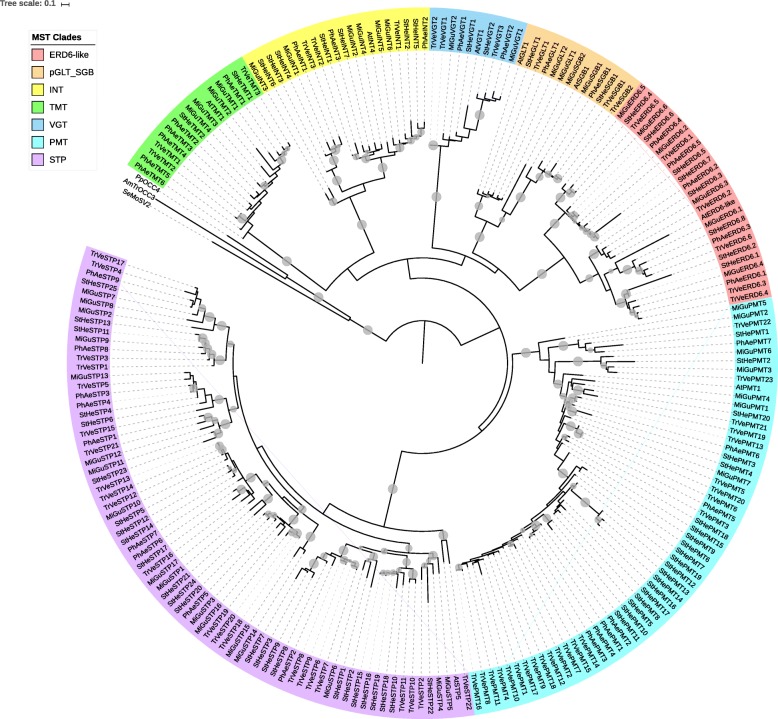
Maximum likelihood tree of the MST genes from parasitic plant transcriptomes and non-parasitic *Mimulus* genome. Groups with average branch length less than 0.025 were collapsed and are shown as gray triangles. In the Figures and Additional Files, bootstrap values greater than 50% are shown as gray circles (15 pixels maximum)

**Fig. 2:**
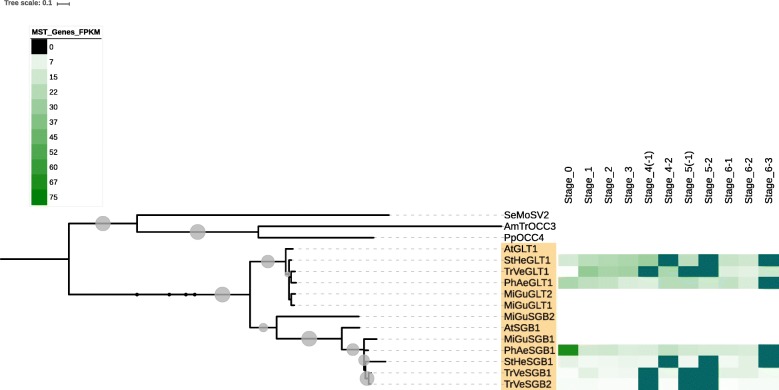
The pGLT clade of MSTs from parasitic plant transcriptomes and non-parasitic *Mimulus* genome, with gene-level expression for members from parasitic plants. Note that for *Phelipanche* and *Triphysaria*, the strongest expression of genes in this clade is usually in stages prior to haustorial attachment. In the heatmaps in the Figures and Additional Files, blank values representing phases not present in a certain parasitic plant are shown as teal blocks

**Fig. 3:**
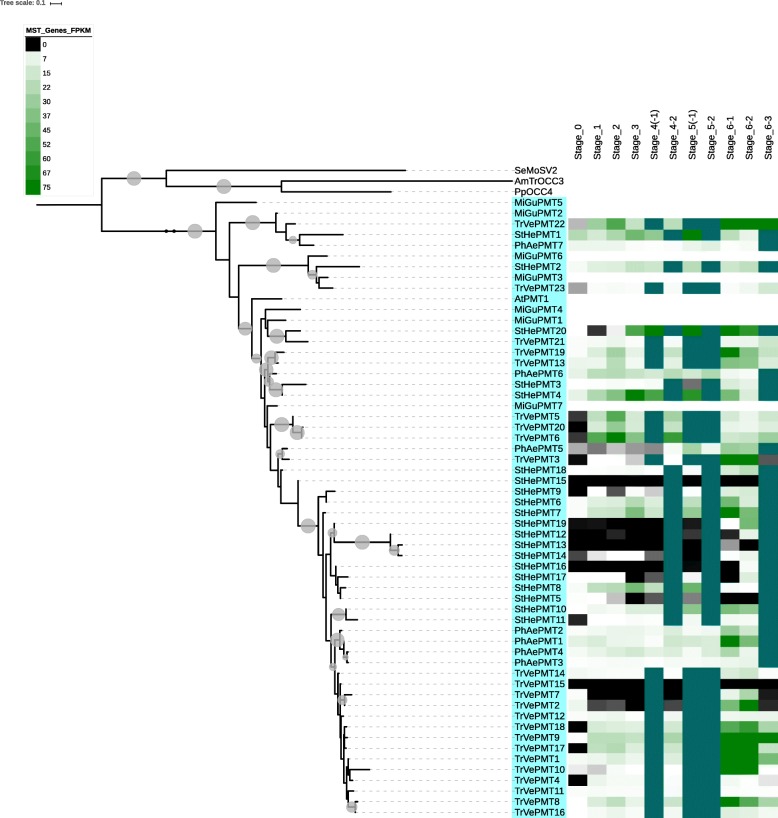
The PMT clade of MSTs from parasitic plant transcriptomes and non-parasitic *Mimulus* genome, with gene-level expression for members from parasitic plants. Note the sharp increase in expression in stage 6–1, the reproductive stage (6–2) and *Triphysaria* stage 6–3

**Fig. 4:**
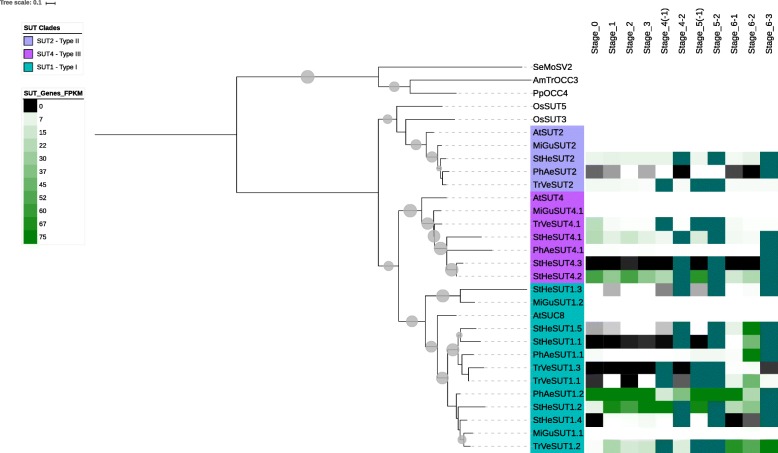
Maximum likelihood tree of the three clades of SUT genes from parasitic plant transcriptomes and non-parasitic *Mimulus* genome. Note the increase in SUT1 expression during stage 6–2 and the heightened expression in *Triphysaria* during germination in the SUT4 clade

**Fig. 5:**
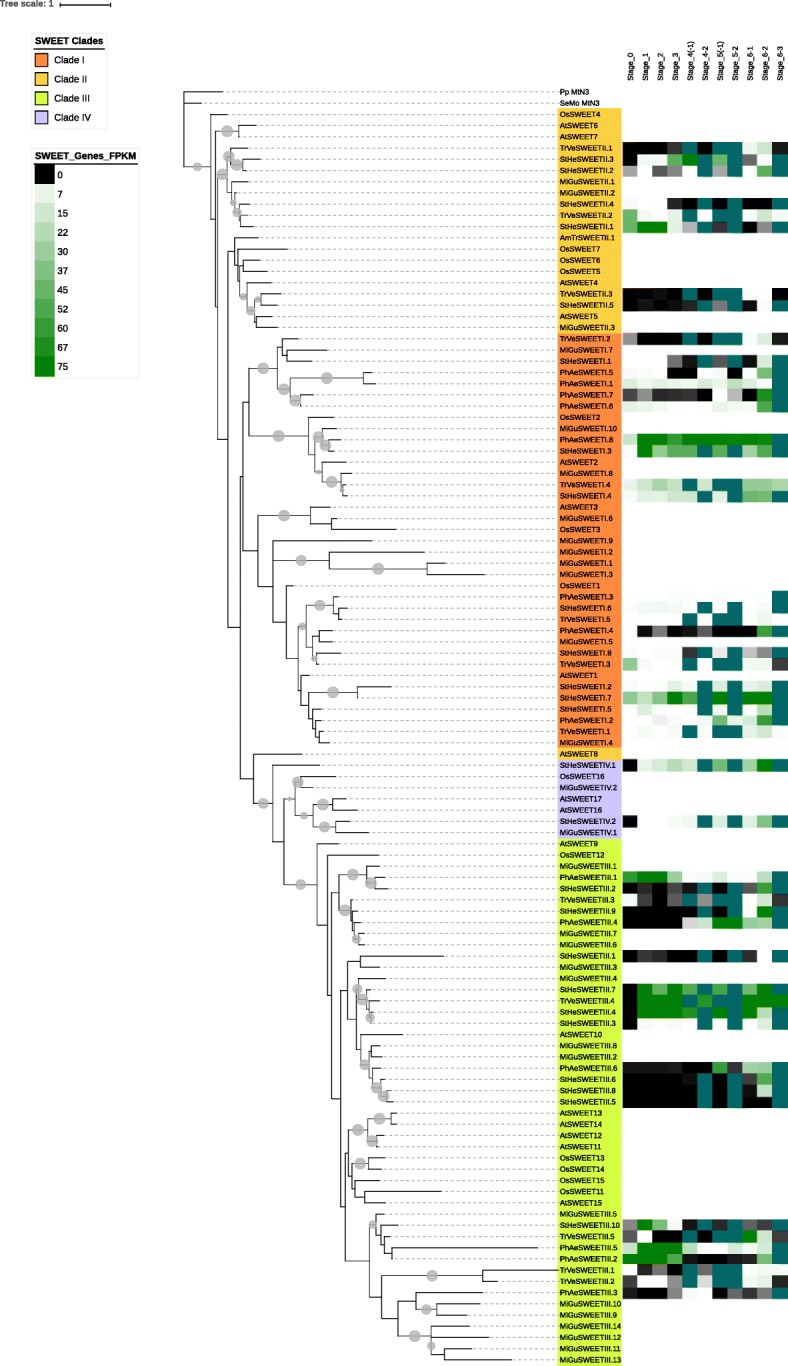
Maximum likelihood tree of the four clades of SWEET genes from parasitic plant transcriptomes and non-parasitic *Mimulus* genome. Note the apparent similarity of the SWEET expression profiles to those of the MST family, as well as the lack of *Phelipanche* sequences in clades II and IV

We next examined the phylogenetic relationships of the various members of the MST, SUT and SWEET gene families within and among the parasitic species in order to determine whether similarities and differences in particular clades account for the observed variation in estimated gene numbers. Significant differences in MST family structure were observed among the three parasitic species (Figs. 1, 2, 3, 4 and 5, Table 1, Additional files 1, 2, 3, 4 and 5). For example, the PMT and STP clades were the largest of the clades comprising the MST family in all three parasites, with membership in the ERD6-like clade also being a significant proportion of the total family size (Table 1). *Phelipanche* had the greatest number of TMT genes, whereas *Striga* had the greatest number of ERD6-like, INT, and STP genes. *Triphysaria* had the greatest number of PMT and VGT genes, while *Mimulus* had the greatest number of pGLT/SGB genes.

In general, the three parasites have comparable numbers of members in the three clades comprising the SUT family (i.e., SUT1, SUT2 and SUT4) of these species. The only exceptions are the expansion of SUT1 and SUT4 genes in *S. hermonthica* relative to its counterparts (Fig. 4, Table 1). *S. hermonthica* has the largest number of SWEET genes among the three parasites (Fig. 5, Table 1) and almost twice in the total number of SWEET genes found in *T. versicolor* and *P. aegyptiaca*. Expansion in *S. hermonthica* appears to principally be in SWEET Clades II, and III. Overall, in comparison to their non-parasitic relative *M. guttatus*, the three parasitic plants are over-represented in clade II and under-represented in clade III SWEETS. Of the parasites, the number and distribution of SWEETSs within clades in *S. hermonthica* is most similar to that of *M. guttatus*. Only *Striga* and *Mimulus* had SWEET genes in clade IV (Table 1, Fig. 5).

### Expression of sugar transporters in three parasitic plants

To uncover differences in sugar transport gene expression that may exist among parasites with different levels of host dependency, we first examined the levels of expression (based upon FPKM data) of the various MST, SUT and SWEET family members at the different developmental stages in each of the parasites. We paid particular attention to any notable difference in expression levels before and following host contact. The results of this analysis are discussed below for each transporter family. Descriptions of each stage of the parasite life cycle can be found in the Materials and Methods. In this analysis, in order to avoid underestimating the expression levels of the various sugar transporters, the gene-level expression was initially considered rather than that of each isoform (as shown in Figs. 2, 3, 4 and 5 and Additional files 1, 2, 3, 4 and 5). However, since the possibility exists that different isoforms are expressed during different stages of the life cycle we also examined both the gene-level and isoform-level expression separately. These values are given in Additional files 7, 8 and 9.

#### MST gene family

In general, expression of MSTs in the various parasitic plants increase from germination to pre-attachment root growth (stages 1 and 2), decreases during haustorial connection, and then increases again during pre- and post-emergence growth and development, anthesis and reproduction (Figs. 2, 3, 4 and 5 and Additional files 1, 2, 3, 4 and 5). Among the MSTs, increased expression is most evident in the PMT and STP clades (Additional file 6). However, the sharp increase in MST expression in the post-emergence growth and reproductive stages evident in *Striga* and *Triphysaria* is not seen in *Phelipanche* (Additional file 6). In *Triphysaria*, MST expression is also high during germination and pre-attachment root growth (stages 1 and 2).

Among the three parasitic plants, the expression profiles of genes in the ERD6-like clade show the most differences (Additional files 1 and 6). For example, in *Phelipanche*, the ERD6-like genes are expressed highly during post-attachment root growth (stage 5–1) and stem and leaf growth stages (stage 6–1) whereas in *Striga*, most ERD6-like genes are strongly expressed during the germination and reproductive phases (Additional files 1 and 6).

Members of the pGLT clade of MSTs similarly show differential expression during the parasite life-cycle (Fig. 2, Additional file 6). In *Phelipanche*, pGLT members tended to be most highly expressed in germination. In *Striga*, orthologous pGLT members are expressed the time of haustorial connection (stage 4–1) and in floral bud development in reproduction (stage 6–2), and in *Triphysaria*, sequences in the pGLT clade expressed most strongly during pre-haustorial root growth. In *Triphysaria*, expression of genes in the INT clade was higher in leaf and stem growth (6–1). In *Striga*, INT genes appear to be more highly expressed during pre-haustorial root growth (stage 1) and reproduction (stage 6–2) (Additional files 2 and 6). Of the plants studied, *Mimulus* had the highest proportion of INT annotated genes, and *Triphysaria*, the lowest.

Expression of representative genes in the TMT clade was highest in the pre- and post-emergent growth stages in *Phelipanche*, and post-emergent growth stages in *Striga* and *Triphysaria* (Additional files 3 and 6). Most PMTs were expressed most strongly during post-emergent growth in all three parasites (Additional file 6). However, in *Triphysaria*, PMTs also expressed strongly in pre-haustorial root growth (Fig. 3 and Additional file 6). In *Striga* and *Triphysaria*, the VGT clade members were found to be more highly expressed during post-emergent tissue growth and reproduction (Additional file 6). However, in *Phelipanche*, expression of VGTs was greatest during haustorial connection and post-emergent root growth (Additional files 4 and 6). Members of the STP clade in all three parasitic plants tended to express most strongly during post-emergent growth and reproduction. In *Triphysaria*, STP expression is also strong in pre-haustorial growth (Additional files 5 and 6), and in *Striga*, STP expression is strongest in pre-emergent growth.

#### SUT gene family

The expression of SUT family members appears to be highest in post-emergent growth stages of the life-cycles of the three parasites (Fig. 4, Additional file 6). This is especially evident in *Striga* and *Triphysaria*. However, significant levels of SUT expression can be observed during germination and pre-haustorial growth in *Striga* and *Triphysaria* (Fig. 4, Additional file 6).

Members in the SUT1 clade exhibit high expression during post-emergent growth stages, with highest levels occurring as plants progressed towards reproduction (stage 6–2). In *Phelipanche*, for example, SUT1 genes expressed most strongly in stages 6–1 and 6–2 (above ground stem and leaf growth and reproduction, respectively) whereas in *Striga* and *Triphysaria*, most SUT1 genes were strongly expressed during reproduction (stage 6–2) (Fig. 4, Additional file 6). SUT2 clade members are expressed during various stages throughout the life-cycle (Fig. 4, Additional file 6), with no strong preference for any one developmental time point. Members of the SUT4 clade appear to be expressed to a greater extent in the pre-haustorial root growth stage 3 and haustorial connection phase 4–2 in *Phelipanche*. In *Triphysaria*, SUT4 expression was strongest during germination (stage 0).

#### SWEET gene family

The expression profiles of the members of the SWEET family are shown in Fig. 5. In general, we observed that members of the SWEET gene families from the three related parasites tended to have similar patterns of expression throughout the parasite life-cycle with *Triphysaria* and *Striga* SWEET family members being most similar (Fig. 5, Additional file 6). Overall, members of the SWEET family are most highly expressed during reproduction and floral bud development (stage 6.2) of parasite development (Fig. 5, Additional file 6). In *Striga* and *Triphysaria*, there are also several SWEET genes that express more strongly during germination and pre-attachment root growth (Fig. 5, Additional file 6).

#### Conserved motifs and Orthogroup analyses

We next examined the nature of the conserved motifs defining members of these various sugar transporters using MEME analysis (Additional files 7, 8, 9, 10, 11, 12 and 13) with the orthogroup most commonly associated with each motif and the sequence for each motif found in the MST, SUT and SWEET gene families. This was done to determine if variations within each clade of MST, SUT or SWEET are associated with differences in expression within these clades, and whether there was a one-to-one correspondence between the gene family definition based on our homology searches and orthogroup definitions. The statistics on the motifs whose presence correlated with certain clades, plants and stages are shown in Additional files 10, 11 and 12; these data are summarized in Additional file 13. Overall, within each clade of sugar transporters, there existed a significant number of motifs that were either exclusive to or strongly represented in certain clades of the MST family.

An orthogroup analysis in OrthoFinder [69] (Additional files 14, 15, 16 and 17) on the Galaxy public server at usegalaxy.org [70] revealed that many orthogroups were unique to one clade, but that certain clades had more than one orthogroup. Here it is important to note that while the orthogroups in the MST and SUT families all contained a Sugar_tr PFAM domain and the SWEET orthogroups all had an MtN3_slv domain, that members of different clades (and therefore orthogroups as well) had different conserved MEME domain architectures (Additional files 10, 11 and 12).

In the MST family, the ERD6-like, pGLT, INT and PMT clades each had two orthogroups that were unique to them. The STP clade had five orthogroups. In the SUT family, orthogroup 1431 contained all members of the SUT1 and SUT4 clades, whereas the SUT2 clade contained only orthogroup 3807. In the SWEET family, clade I was almost entirely represented by three orthogroups (with one *Mimulus* SWEET in a fourth orthogroup, 5750), all of which were unique to that clade, all clade II members were part of orthogroup 769, clade III members were part of orthogroup 429, and clade IV members belonged to orthogroup 2089.

In several instances different orthogroups within the same clade were found to have their highest levels of expression at different developmental stages. An example where this can be seen is the five orthogroups of the STP clade (Additional file 17). This may be explained by the fact that different STP clade members can have varying affinities for different monosaccharides [45].

## Discussion

Parasitic weeds rely upon their host to different extents for their nutritional needs and therefore the organization and expression of nutrient transporters would likely need to vary both developmentally and temporally during their life cycle. Our findings indicate that members of the *Orobanchaceae*, while sharing sugar transporter genes in common, may adjust the expression of these genes in response to their life-styles. *P. aegyptiaca* is a holoparasite and *S. hermonthica* is an obligate hemiparasite [27]. Since holoparasites have lost anatomical and molecular features associated with photosynthesis [27, 30, 70, 71], it is expected that sugar transporters associated with photosynthesis would be lost in *Phelipanche*. The difference in repertoire size between *Phelipanche* and *Striga* (i.e., *Phelipanche* has fewer MSTs, SUTs and SWEETs than *Striga*) could reflect the differential state of host dependency in the parasites since a number of the sugar transporters associated with photosynthesis may be lost in *Phelipanche*. Differences in the composition of the MST, SUT and SWEET families among the parasites might also reflect differences in nutrient preference between holoparasitic, obligate and facultative hemiparasitic forms [72].

### Phylogenetic Analysis of Sugar Transporter Gene Families

The three parasitic plants transcriptomes examined contained representatives of all seven clades of MSTs present in plants (i.e., ERD6-like, pGLT/SGB, INT, TMT, PMT, VGT, STP), three SUT family clades associated with dicots (i.e., SUT1, SUT2 and SUT4), and the four clades of SWEETs previously identified. We queried the datasets for all three parasites to determine whether any orthologs of the two monocot specific clades of SUTs defined by Kuhn & Grof [46] were not present since there is a possibility of their presence resulting from horizontal gene transfer (HGT) as described in *S. hermonthica* and other plants in the *Orobanchaceae* family [73–76]. However, we did not find any SUTs that were nested within with members of SUT3 or SUT5 (see Fig. 4 and Additional file 8).

The parasitic plant transcriptomes studied had a lower proportion of members in the ERD6-like clade than observed in the *A. thaliana* genome, which has 19 members accounting for 35.8% of all *Arabidopsis* MSTs [77]. Since ERD6-like proteins transport glucose from vacuole to cytoplasm in non-parasitic plants [78], and taking into account that parasitic plants have been suggested to be strong sugar sinks [65], it is possible that ERD6-mediated glucose transport may be limited in parasitic plants. Since there is evidence that some STPs function preferentially in root and pollen development [45], it is not unreasonable to speculate that differences in the STP clade sizes among the three parasitic plants could reflect differential needs in each plant.

It has been previously reported that SWEET13 homologs in maize has stronger expression in leaf vasculature and thus may be important in photosynthetic carbon movement and that SWEET13a, b, c triple-knockout mutants have impaired photosynthesis [76]. In this study, we observed that expression of genes in *Phelipanche* SWEET Clade III is stronger in stages of growth that do not require photosynthesis (i.e., Stage 5–2 and before pre-emergence shoot growth) (see Additional file 6). In contrast, *Striga* and *Triphysaria*, show stronger expression of SWEET clade III in the post-emergent stages 6–1 (leaves and stems) and reproductive Stage 6–2, and floral structure development in stage 6–3. Because *Striga* and *Triphysaria* are capable of photosynthesis, unlike the holoparasitic *Phelipanche*, the difference in expression levels between *Phelipanche* and the hemiparasitic *Striga* and *Triphysaria* appear to be an example of differences in sugar transporter activity between parasitic plants correlated with their different degree of host dependence.

We also found that *Phelipanche* had the highest proportion of clade I SWEETs (57.14% of SWEETs in *Phelipanche*) (Table 1), that *Phelipanche* and *Triphysaria* lacked any clade IV SWEETs, and that parasitic plants smaller proportion of clade III SWEETs than in *Mimulus*, while *Striga* and *Triphysaria* have a greater proportion of clade II SWEETs than *Mimulus*. Together, these results suggest that overall; the SWEET family of sugar transporters in the *Orobanchaceae* family of parasitic plants may be organized differently from other plants. However, it must be noted that the transcriptomes studied contain genes that have sufficiently high transcription levels. There may be genes in these sugar transporter families that are not transcribed at high enough levels to be included in the transcriptomes studied, but may be found should full genome assemblies and annotations become available.

### Expression of sugar transporters in three parasitic plants

According to Figs. 2 and 3 and Additional files 1, 2, 3, 4 and 5 and 7, all parasitic plants in this study in general increase in MST expression during pre-haustorial root growth phases, decrease during haustorial attachment and penetration, and increase significantly during pre- and post-emergent developmental phases. This result is consistent with most damage being done to the host plant during post-connection growth stages [1]. Given the findings that carbohydrates accumulate in pre-emergent shoots in *P. aegyptiaca* [79], it is possible that sugar transporters could be involved in mobilizing the storage of these carbohydrates during the flowering phase.

Some ERD6-like proteins have been shown to be involved in the transport of sugars out of vacuoles [78]. During the post-emergent growth and reproduction stages of the parasites there is likely a greater need for host nutrients and therefore it might be expected that as the parasitic develops, some ERD6-like proteins would be differentially required based on individual parasite needs. It has been previously reported that INTs are localized in the tonoplast and play important roles in root development [43]. In this current study, the non-parasite *Mimulus* was found to have a high proportion of INTs compared to its parasitic relatives, and the facultative *Triphysaria* had an especially low proportion of INTs, suggesting that parasitic plants may have different requirements for INTs than their non-parasitic counterparts. In *Striga*, TMT expression was more likely to express strongly during vegetative growth stage 6–1, and in *Phelipanche*, pre-emergent root growth showed the greatest likelihood for strong TMT expression (Additional files 6 and 7), suggesting higher mobilization of glucose and fructose to the vacuolar lumen in these plants, similar to what is seen in *Arabidopsis thaliana* [80]. The profile of polyols is host dependent [81]. For example, *P. aegyptiaca* accumulates mannitol when on a tomato host [82]. PMTs, along with VGTs, are known for long-distance transport and phloem loading [83]. The parasites investigated in the present study were all grown on different hosts and, therefore, it is not surprising that the PMT genes have different expression profiles in the different parasitic plants. If one assumes that plants metabolize different polyols, and accumulate different levels of various polyols in various tissues [84] then one might expect varied levels of transporters throughout the lifecycle. The findings in this study (see Additional file 6) suggest that members of the PMT clade were most likely to exhibit high level of expression in stages 6–1 through 6–3. In *Arabidopsis*, VGT proteins are mainly expressed in above ground tissues [85]. The observed high expression of VGT genes in post-emergent growth stages, especially vegetative growth (stage 6–1) in *Striga* and *Triphysaria* is consistent with these findings. The expression of groups of STPs in growth and reproductive stages, especially in *Striga* and *Triphysaria*, is consistent with STPs in *Arabidopsis* being expressed during pollen development and root development [45].

Weise et al. [53] reported that SUT4 is associated with movement of photosynthates whereas Frost et al. [54] reported that SUT4 is involved in transport process associated with water stress. In the present study, SUT2 was the weakest expressed member of the SUT family in the parasitic plants (Fig. 4, Additional file 8) consistent with the results of Peron et al. [65], who reported that SUT2 transcripts accumulate at low levels throughout the *P. ramosa* life cycle*.* In contrast, expression of *PrSUT1* and *PrSUT3*, (members of the SUT1 clade in *P. ramosa*), were expressed highest after emergence of the flowering shoot [65]. We similarly observed SUT1 expression to be strongest during reproduction in the three parasites.

In non-parasitic plants, SUT1 is associated with phloem loading [51, 52, 86] and unloading [50] and SUT2 has been proposed to be a sucrose sensor [52]. Thus, it is possible that in *Triphysaria*, a facultative hemiparasite, the mechanisms for nutrient movement in the free living state are similar to those for nutrient movement when attached to a host. It is also possible that SUT1s are involved in retrieval of sucrose during transport in sieve elements and unloading in sink organs such as roots and flower structures. The expression data in Fig. 4 and Additional file 8 suggest sucrose unloading into flower buds. To fully understand whether this is the case, though, studies on regulation of and by SUT1 and SUT2 in parasitic plants would have to be done.

In this study, SWEET expression, especially in *Striga* and *Triphysaria*, were strongest during post-connection growth and especially reproductive stages. These results are thus consistent with the finding that SWEETs have been shown to efflux sucrose out of parenchyma cells into the phloem [35]. However, there were members of SWEET clades II and III in *Triphysaria* that express more strongly in pre-host attachment stages. Given the growth of roots and haustoria in these stages [28] and given the role of clade III SWEETs AtSWEET11 and AtSWEET12 in root growth [35, 64], it is possible that SWEETs have roles in pre-haustorial root growth in parasitic plants such as *Phelipanche* or *Triphysaria*.

Fig. 2, 3, 4 and 5 and Additional files 6, 7, 8 and 9 suggest the presence of few instances of high expression during haustorial attachment (stage 3–4). While it is possible that expression shifting among the members of the sugar transporter families could be significant, it is important to note that were it available, careful annotation of a full genomic assembly could identify additional undetected orthologous genes or nearly identical paralogous genes that are not distinguishable in de novo transcriptome assemblies

The movement of RNAs (both transcripts and small RNAs) has been reported to occur between hosts and parasitic plants [87, 88]. Therefore, it may be possible to use transgenic expression of RNAi to reduce or eliminate specific SWEETs necessary for post-attachment development of host-parasite connections required for parasite nutrient acquisition. The use of RNAi to better defend host plants from parasitic plants has been proposed [89], and suggestions that this approach may be successful have appeared see for example refs. [90, 91]. Whether or not such approaches would eventually also succumb over time allowing the parasite to bypass the need for specific targeted transporters remains an open question [92].

### Conserved motifs and Orthogroup analyses

In this study, MEME analyses [93] identified conserved motifs in common and unique to proteins encoded by members of genes in different clades, such as the ERD6-like and PMT clades (see Additional files 10, 11, 12 and 13). These conserved motifs may be essential to the different functions of the encoded proteins (e.g., their cellular targeting or substrate utilization) corresafponding to the differential developmental and temporal expression of genes within these clades. In addition, there exists a significant number of motifs in the SUT and SWEET families that were exclusive to *Arabidopsis*, rice and the outgroup genes in this study. These results suggest that parasitic plant sugar transporters are likely to use different motifs from those in non-parasitic plants suggesting that protein structural difference may have evolved to fulfill specialized functions in the parasite.

In addition, consistent with expectations, clade definition and orthogroup definition were different. While some clades only had one orthogroup, other clades consisted of multiple orthogroups. In the MST family, while there were no instances of one orthogroup being in multiple clades, there were instances of one clade having more than one orthogroup (Additional file 16).

In the SUT family of parasitic plants, all SUT1 and SUT4 sequences are associated with the same orthogroup, a result consistent with phylogeny proposed by Peng et al. [58] in which SUT1 and SUT4 are classified as members of the same Ancient Group (AG1). The orthogroup composition of the sugar transporter gene families was used to investigate whether orthogroups could explain variations in expression within a clade. As expected, different orthogroups within a larger clade showed differential expression preference for different stages of the life cycle (e.g., in the STP clade). However, some of this variation may be explained by members of different orthogroups being more frequent in different species (Additional file 17). Of the seven orthogroups identified in the SWEET family, only clade II (orthogroup, group 769) and clade IV (orthogroup 2089) had single orthogroup membership (Additional file 16). Clade I and II however shared some orthogroup overlap indicating a possible relatedness between these clades.

The above results underscore the need for further studies to determine how the members of the different clades of MST, SUT and SWEET transporters are used during parasite interactions with the host. While examining conserved protein motifs represented in the various MST, SUT and SWEET transporters may serve as one level of analysis, it might also be enlightening to look at the conserved regulatory motifs in the promoters of these various genes responsible for their differential expression. However, at the present time only transcriptomic data are available for the parasitic plants studied here, and therefore, such an analysis cannot be done until whole-genome data becomes available.

## Conclusions

In this study, we identified members of the MST, SUT and SWEET families of sugar transporters in three parasitic weeds, *P. aegyptiaca*, *S. hermonthica*, and *T. versicolor*, and investigated the phylogenetic relationships among the various gene family members and their differential expression during parasite growth and development. We showed that members of different clades of MSTs, SUTs and SWEETs were differentially expressed throughout the parasitic plant life cycle and expression profiles were dependent on the parasitic plant. Our observations indicate that that different parasitic plants are regulating sugar transporter expression differently and speculate that some of these differences may be due to the differences in host species and degree of host dependency exhibited by the parasitic plant species. This is consistent with prior suggestions that parasitic plants may change the expression of genes within their regulatory networks to most effectively parasitize a host. How this is accomplished remains unknown, but could involve the differential expression or activation of members of transcription factor and transcriptionally-active protein gene families that regulate sugar transporter gene expressions as previously proposed [32, 94]. These studies do however identify potential targets for directed manipulation that will allow for a better understanding of the nutrient transport process and perhaps a means for controlling the devastating effects of these parasites on crop productivity.

## Materials and methods

### Tissues, transcriptomic library preparation and sequence analysis

Detailed descriptions of parasite growth and the collection of biological materials from the parasite developmental stages (which extend from imbibed seed, germination and haustorial development, attachment to above ground tissues) can be found in Westwood et al. [28]. Transcriptome sequencing was previously described in Yang et al. [31] and encompassed multiple stages of parasite development from the species *T. versicolor*, *S. hermonthica*, and *P. aegyptiaca* within *Orobanchaceae* as described therein.

### Data sources

Transcriptome assemblies and expression data for *P. aegyptiaca*, *S. hermonthica,* and *T. versicolor* were obtained from the PPGP II datasets from the PPGP Website (http://ppgp.huck.psu.edu/download.php). The de novo assemblies were performed with Trinity [95] and post processed into non-redundant sets including predicted coding sequences and their corresponding using the PlantTribes pipeline (https://github.com/dePamphilis/PlantTribes) [31]. Cleaned reads were mapped to post-processed assembled transcripts and expression abundance of the parasite developmental stages [28] estimated using the RSEM pipeline [96] with the Bowtie2 [97] read aligner option. A detailed description of the PPGP II datasets is available on the PPGP website.

Known *Arabidopsis thaliana* and rice SWEET sequences were retrieved from the NCBI Protein, Nucleotide, and EST Databases [98–100]. The *Arabidopsis* MSTs and SUTs used for each clade (from [38, 41]) were also found on those same databases. The *Mimulus guttatus* genome version 2.0 [101, 102] was retrieved from Phytozome version 12.1 [103, 104].

### Identification of sugar transporters

The *Arabidopsis* sugar transporter sequences and rice SWEET sequences that were found as described above were used as queries to search for potential sugar transporters in the assembled transcriptomes of the parasitic plants *P. aegyptiaca*, *S. hermonthica,* and *T. versicolor*, as well as the annotated genome of the related non-parasitic plant *Mimulus guttatus*. In searching for sugar transporter sequences, the FASTA, FASTX and TFASTX methods [105, 106] from FASTA version 36.3.8e (September 30, 2016, accessed from https://fasta.bioch.virginia.edu/fasta_www2/fasta_list2.shtml [107]) was used with an E-value cutoff of 1e-3. The potential sequences were retrieved using custom Perl scripts that incorporated modules from BioPerl 1.7.1 [108, 109]. Any coding sequence (CDS) or nucleotide (NT) sequences were translated into amino acid sequences across six reading frames using a custom BioPerl script [110]. The sequences were searched using SANSParallel [111, 112] against the UniprotKB database [113], with 50 hit sequences displayed per query, and the “very slow” setting. For all BLAST results, inspection of best matches for each query sequence was used to determine if a sequence was a plant MST, SUT or SWEET. Only sequences whose best matches had an E-value of 1e-3 or smaller were kept for further analyses.

### Curation, multiple sequence alignment, and phylogenetic analysis

MEME Suite 4.12.0 [93, 114] command line was used to discover motifs in all sugar transporter sequences studied, with any number of repetitions expected, 100 motifs maximum, and each motif having a width ranging from 25 to 250 amino acids long, and maximum iterations at 250. All species were analyzed in the same MEME run in order to accurately determine which motifs sugar transporter sequences had in common, and which motifs, if any, may be essential to the function of members of certain clades of the MST, SUT or SWEET families of sugar transporters. In addition, the discovered motifs were used to curate all sequences analyzed and eliminate false positives in the sequence sets, thus ensuring more accurate downstream analyses. MEME was used twice in this study, once as a tool for curation, and the second time as a tool to compare sequences within a gene family (described below).

Multiple sequence alignment was done using MAFFT L-ins-i version 7.312 [115, 116].

MSTs and SUTs are members of the MFS superfamily and contain Sugar_tr or MFS_1 PFAM domains, and SWEETs contain the MtN3_slv PFAM domain. Therefore, sequences with the appropriate PFAM domains (Sugar_tr and MFS_1 for both MST and SUT, GPH_sucrose for SUT, and MtN3_slv for SWEET) were used to aid in rooting sugar transporter phylogenetic trees; these sequences were from the *Physcomitrella patens* genome assembly v3.3 [103], the *Selaginella moellendorffii* assembly v1.0 [117], and the genome of the basal angiosperm *Amborella trichopoda* v1.0 [118]. For MSTs and SUTs, a keyword search using the term “MFS” in Phytozome was used. For the SWEETs, rooting sequences were found by using BLASTP against the Phytozome Database [103] with an E-value cutoff of 1e-5, using *Physcomitrella patens* sequences from the OrthoMCL database (ppat|e_gw1.127.40.1, orthogroup OG5_127038) [119]; the best hits from each of the three proteomes searched were kept as sequences to be used as outgroups for the SWEETs in this study.

Sequence sets for proteins and transcripts of the genes studied were analyzed. First, transcript sequences were aligned and examined for isoforms, the method for which is described below. Once isoforms were removed from the set, the names of the remaining transcript sequences were used to retrieve their protein counterparts. The proteins were then aligned and examined for isoforms. Isoforms of the same gene for *Mimulus*, *Arabidopsis* and rice sequences were removed prior to multiple sequence alignment. Parasitic plant sequences that were potentially isoforms of the same gene were eliminated from the sequence set as described below. To ensure an accurate phylogenetic tree, trimAl 1.4.1 [120] was used to first trim alignments to remove columns with less than 10% occupancy, and then eliminate any sequences that covered less than 50% of the alignment (−resoverlap 0.50 –seqoverlap 50).

The alignment process at both the transcript and the protein levels consisted of using a BioPerl script we developed to eliminate redundant sequences, running MAFFT, and then running trimAl. This process was repeated until all sequences covered at least 50% of the alignment. A similar procedure has been used in Yang et al. (2015) [31]. In addition, to find potential outliers in the phylogenetic dataset, a preliminary neighbor-joining (NJ) phylogenetic tree was made with ClustalW2 program [121], using uncorrected p-distance and no gap exclusion. If any non-outgroup branch length for a sequence was shown to be unusually long (i.e., branch length > 0.3 when related branches had lengths of under 0.1), the unaligned, non-trimmed version of that sequence was retrieved from the unedited assembly and then manually curated using information from SANSParallel, MEME or the Conserved Domain Database [60, 93, 122]. After that, the newly edited version of the sequence replaced the non-edited version in the set of sequences to align. Isoforms of the same gene were found and eliminated from the sequence set in the preliminary NJ tree mentioned above for sequences with one or more of the following criteria: high sequence similarity (indicated by branch lengths < 0.01), length of sequences (too short and the sequence was suspected to be a truncated version of the gene), and similarity of MEME protein motif architecture. Then the sequences were re-aligned. The process of detecting long branches, curating sequences on long branches, removing potential isoforms, and re-aligning was repeated until the presence of very long branches and isoforms, which could mislead phylogenies, was minimized or eliminated. The resulting curated set of sequences was used in phylogenetic analysis. Phylogenetic trees were made using RAxML 8.2.11 [123, 124], using 1000 bootstrap replicates and using the PROTGAMMAAUTO option, which automatically includes model testing, with five gamma categories, and corrected Akaike information criterion (AICc) [125]. The names of the sequences on the phylogenetic trees in the Figures and Additional files are shorthand names, and are listed in Additional file 18 alongside their corresponding names from the FASTA files.

### Analysis of conserved motifs and Orthogroups

The names of the sequences that were phylogenetically analyzed were used to retrieve the unedited versions of the sequences (i.e., the versions of the sequences prior to curation and alignment). These unedited versions of the sequences were run through MEME (using the parameters described above). The resulting motifs were included in Additional files 7, 8 and 9. To avoid the presence of identical gene copies, which would have made the analyses less accurate, the sequences that were eliminated during curation and alignment were not included in this analysis. The MEME domains were linked to the sequences to which they belonged in order to determine which clade, life cycle stage, and species with which the MEME motifs were most strongly associated.

The set of unedited versions of the sequences used in the phylogenetic analysis were also used in an OrthoFinder [68] analysis on the Galaxy server [70], using both blastp and hmmscan as protein classifiers, and a minimum e-value of 1e-5.

### Analyses of differential gene expression

Expression values for the parasitic weeds upon infecting a host [31] were based on read counts expressed as FPKM (fragments per kilobase of unigene length per million reads), which were based on the results of read mapping for every gene in the library for each species, after parasitic weeds were allowed to infect host plants. For each gene, each FPKM value in the data studied here represents the sum of FPKM values from all isoforms for a given gene. That is, after *Phelipanche* infected *Arabidopsis thaliana* and tobacco; after *Striga* infected sorghum; and after *Triphysaria* infected *Medicago truncatula* [28, 32].

The names of each developmental stage in the parasite are described in detail in Westwood et al. (2012) [28] and illustrated in Yang et al., (2015) [31]. In the present study, stage 0 represents germination, 1 and 2 represent radicle elongation and haustorial differentiation, respectively [28]. Stage 3 represents the phases in which a parasitic plant uses its haustoria to attach to the host plant, and in stage 4, the parasitic plant connects to its vascular system [28]. Stage 5 is the phase of the growth of pre-emergent tissues, roots (5.1) and shoots (5.2). Stage 6.1 represents the post-emergent growth of leaves and stems, and stages 6.2 represented reproduction, the development of floral buds, while stage 6.3 represented further floral maturation in *Triphysaria* [28].

The expression values for each sugar transporter sequence were placed into a text file using a custom Perl script that we developed. The phylogeny, expression data and conserved MEME domains were visualized using Interactive Tree of Life (iTOL) version 4 [126, 127].

## Additional files

Additional file 1: The ERD6-like clade of MSTs from parasitic plant transcriptomes and non-parasitic *Mimulus* genome. Please note the *Triphysaria* ERD6-like clade members with strongest expression in stage 2, in contrast with *Phelipanche* and *Striga*. (PDF 30 kb)

Additional file 2: The INT clade of MSTs from parasitic plant transcriptomes and non-parasitic *Mimulus* genome. Note that, as with most other parasitic plant sugar transporter families, there is an increase in expression in pre-haustorial growth stages, a decrease in expression during haustorial connection, and an increase in expression in pre- and post-emergence growth and reproduction. (PDF 26 kb)

Additional file 3: The TMT clade of MSTs from parasitic plant transcriptomes and non-parasitic *Mimulus* genome. Note the slight increase in TMT expression during pre-connection and haustorial connection phases. (PDF 24 kb)

Additional file 4: The VGT clade of MSTs from parasitic plant transcriptomes and non-parasitic *Mimulus* genome. Note the tendency to express more strongly in stages 6–1 and 6–2. (PDF 23 kb)

Additional file 5: The STP clade of MSTs from parasitic plant transcriptomes and non-parasitic *Mimulus* genome. Note the increase in expression during post-attachment stages in *Triphysaria*. (PDF 44 kb)

Additional file 6: Numbers of sugar transporters and their stages of strongest expression in *Phelipanche aegyptiaca.* The stages of strongest expression are shown for each clade of MSTs, SUTs and SWEETs, as well as for all MSTs, SUTs and SWEETs, in the transcriptomes of *P. aegyptiaca*, *S. hermonthica*, *T. versicolor*. (XLSX 13 kb)

Additional file 7: Maximum likelihood tree of the MST genes, with heat map and domain architectures. Note the general increase in expression with each successive stage of the life cycle. (PDF 158 kb)

Additional file 8: Maximum likelihood tree of the SUT genes, with heat map and domain architectures. Note that in the SUT1 clade, there is a tendency for genes to express more strongly in later life cycle stages, culminating in a sharp increase in expression in stage 6–2. (PDF 43 kb)

Additional file 9: Maximum likelihood tree of the SWEET genes, with heat map and domain architectures. Note the strong tendency to show higher levels of expression during the reproductive stage 6–2, with some exceptions in clades II and III. (PDF 73 kb)

Additional file 10: MEME motifs and their associations with stages of the life cycle. These associations were determined by finding the genes to which these motifs belonged, and determined the stage in which these genes are most strongly expressed. This was done for (A) MSTs, (B) SUTs, and (C) SWEETs. The motifs most strongly associated with given stages were determined by finding the stage with the highest raw number of occurrences of the motif in that stage, as well as the representation of that motif as a percentage of motifs present in genes that showed strongest expression during that stage. (XLSX 47 kb)

Additional file 11: MEME motifs and their associations with plants studied. These associations were determined by finding the genes that contain a given motif. This was done for (A) MSTs, (B) SUTs, and (C) SWEETs. The plant species with which motifs are most strongly associated was determined by finding the plant with the highest raw number of occurrences of the motif, as well as the representation of that motif as a percentage of instances of that motif in the MST, SUT or SWEEET family. (XLSX 33 kb)

Additional file 12: MEME motifs and their associations with clades of MSTs, SUTs or SWEETs. These associations were determined by finding which genes to which these motifs belonged, and determined the clade in which these genes belonged. This was done for (A) MSTs, (B) SUTs, and (C) SWEETs. The clade with which motifs are most strongly associated was determined by finding the clade with the highest raw number of occurrences of the motif in that stage, as well as the representation of that motif as a percentage of motifs present in genes that showed strongest expression during that clade. In addition, MEME motifs unique to MST, SUT or SWEET clades were found and counted. (XLSX 43 kb)

Additional file 13: MEME motifs in the (A) MST, (B) SUT and (C) SWEET families of sugar transporters. For each motif, the stage in which the genes containing these motifs express most strongly is shown, as well as the plant and clade in which genes containing these motifs are most commonly found, and the consensus sequence for each motif. (XLSX 24 kb)

Additional file 14: Orthogroups in each stage of three *Orobanchaceae* life cycles. The data suggest different orthogroups being expressed in a variety of stages throughout the life cycle, especially in the MST family. (XLSX 21 kb)

Additional file 15: The species to which orthogroups are most frequently associated. Note the differences between the raw number of members in a certain orthogroup versus the percentage of sequences from a species that are members of that orthogroup. (XLSX 13 kb)

Additional file 16: The clades with which each orthogroup is most strongly associated. These orthogroups were identified in the A) MST, B) SUT and C) SWEET families. Note that many sugar transporter clades have at least one orthogroup that is unique to them. In the SUT family, one orthogroup has all SUT1 and SUT4 sequences studied as members. (XLSX 14 kb)

Additional file 17: Every sugar transporter gene, their strongest stage of expression, clade and orthogroup. The clade, stage of strongest expression and orthogroup are listed for genes in the A) MST, B) SUT and C) SWEET families. (XLSX 10 kb)

Additional file 18: The names of the genes on the phylogenetic trees, and their FASTA file aliases. The names as they appear in Figs. 1, 2, 3, 4 and 5 and in Additional files 1, 2, 3, 4, 5 and 7, 8, 9 are shorthand labels that show the species (e.g., *Striga hermonthica* is shortened to StHe), the clade, and an arbitrarily given number. (XLSX 17 kb)

## Abbreviations

AG1: Ancient Group 1
AG2: Ancient Group 2
CDD: Conserved Domain Database
CDS: Coding sequence
CDS: Coding sequence
EBI: European Bioinformatics Institute
ERD6-like: Early Response to Dehydration 6-like
ESL: Early Response to Dehydration 6-like
EST: Expressed sequence tag
FPKM: Fragments per kilobase of unigene length per million reads
GO: Gene Ontology
HGT: Horizontal gene transfer
INT: Inositol transporter
iTOL: Interactive Tree of Life
MAFFT: Multiple Alignment using Fast Fourier Transform
MEME: Multiple Em for Motif Elicitation
MFS: Major facilitator protein
MST: Monosaccharide transporter
MtN3: *Medicago truncatula* nodulin 3
NT: Nucleotide
NT: Nucleotide
pGLT: Plastidic glucose translocator
PLT: Polyol transporter
PMT: Polyol/monosaccharide transporter
PPGP: Parasitic Plant Genome Project
RAxML: Randomized Axelerated Maximum Likelihood
RNAi: RNA interference
SANSParallel: Suffix Array Neighborhood Search Parallel
STP: Sugar transport protein
Sugar_tr: Sugar transporter
SUT: Sucrose transporter
SWEET: Sugars will eventually be exported transporters
TM: Transmembrane
TMT: Tonoplastic monosaccharide transporter
TST: Tonoplastic sugar transporter
VGT: Vacuolar glucose transporter
