# Supplementary figures and images for: Genome-wide identification of MST, SUT and SWEET family sugar transporters in root parasitic angiosperms and analysis of their expression during host parasitism
Source: BMC Plant Biol. 2019 May 14;19:196. doi: 10.1186/s12870-019-1786-y (PMC6515653; doi:10.1186/s12870-019-1786-y)

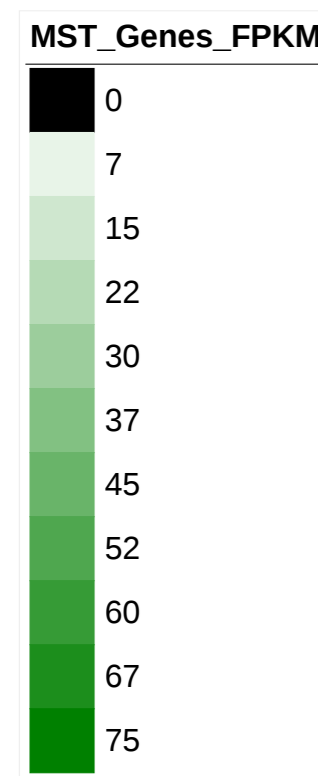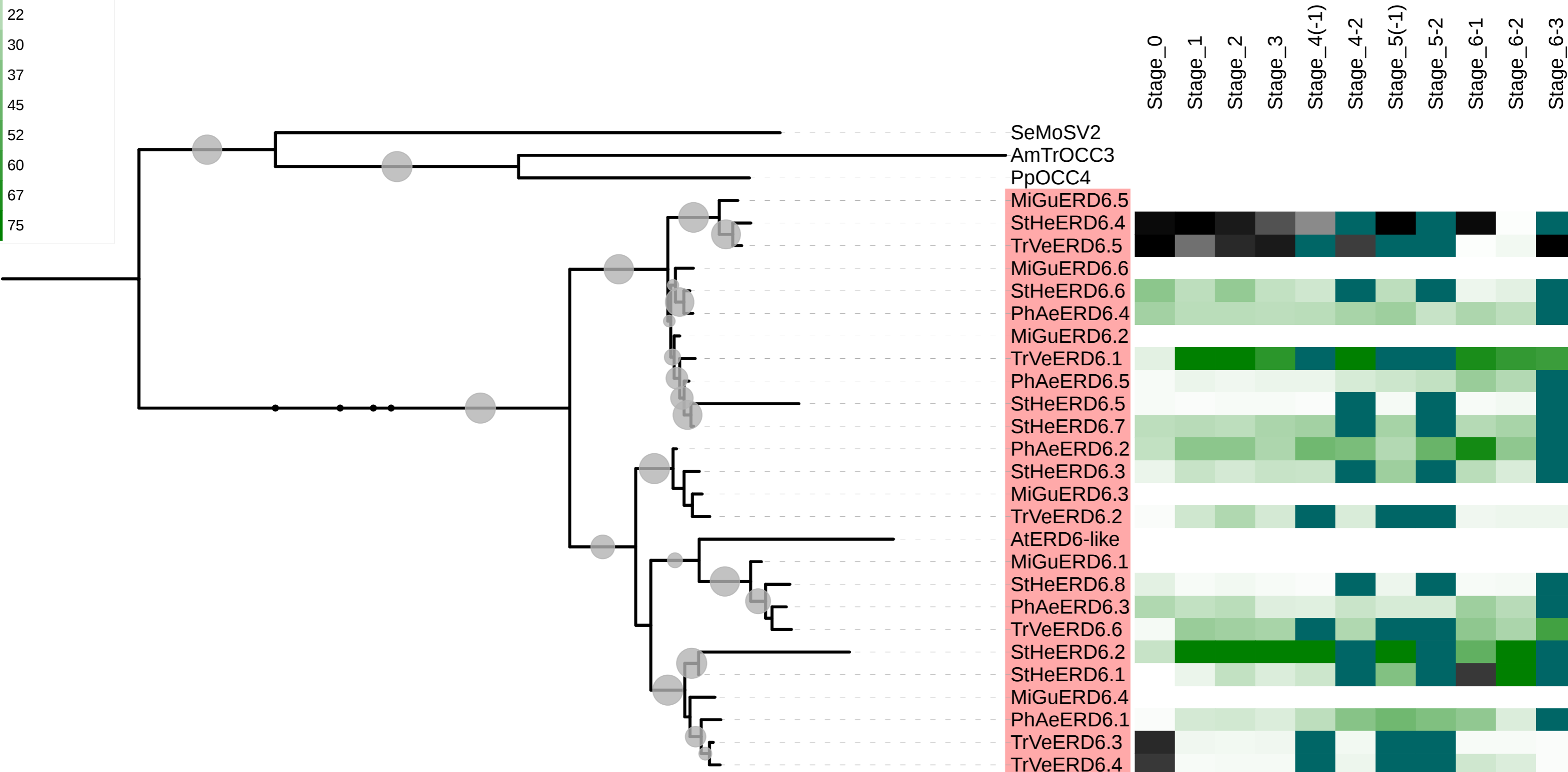

Supplement: Supplementary file 1 — The ERD6-like clade of MSTs from parasitic plant transcriptomes and non-parasitic Mimulus genome. Please note the Triphysaria ERD6-like clade members with strongest expression in stage 2, in contrast with Phelipanche and Striga. (PDF 30 kb) [file 12870_2019_1786_MOESM1_ESM.pdf]

Tree scale: 0.1

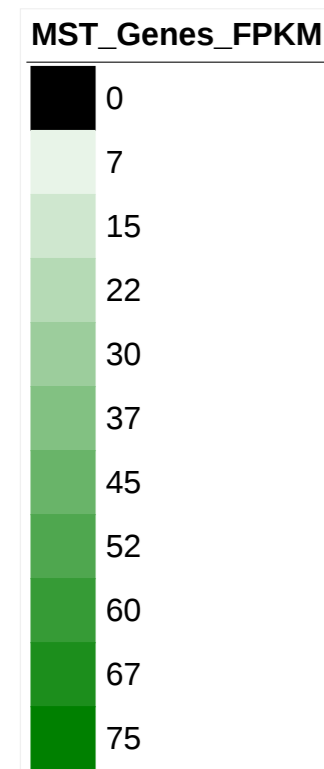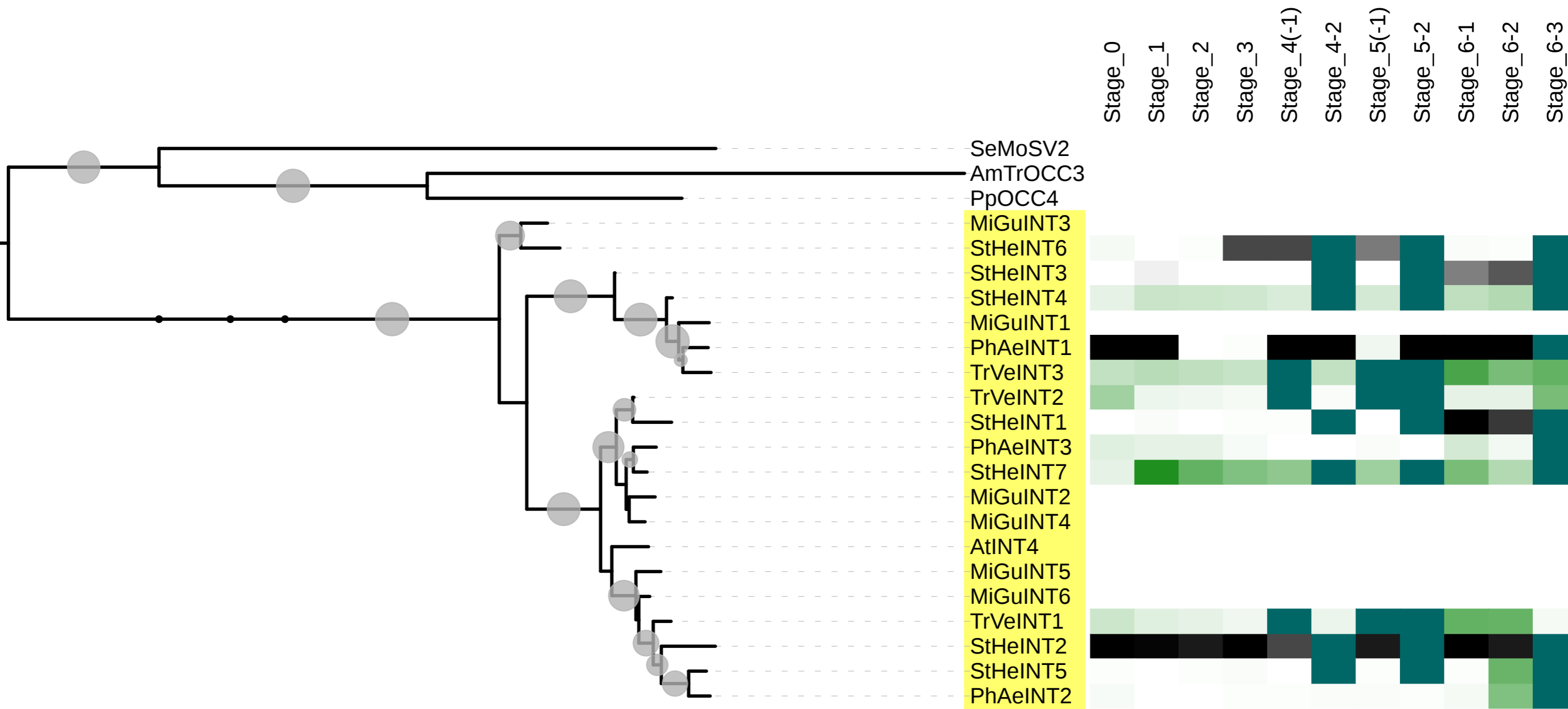

Supplement: Supplementary file 2 — The INT clade of MSTs from parasitic plant transcriptomes and non-parasitic Mimulus genome. Note that, as with most other parasitic plant sugar transporter families, there is an increase in expression in pre-haustorial growth stages, a decrease in expression during haustorial connection, and an increase in expression in pre- and post-emergence growth and reproduction. (PDF 26 kb) [file 12870_2019_1786_MOESM2_ESM.pdf]

Tree scale: 0.1

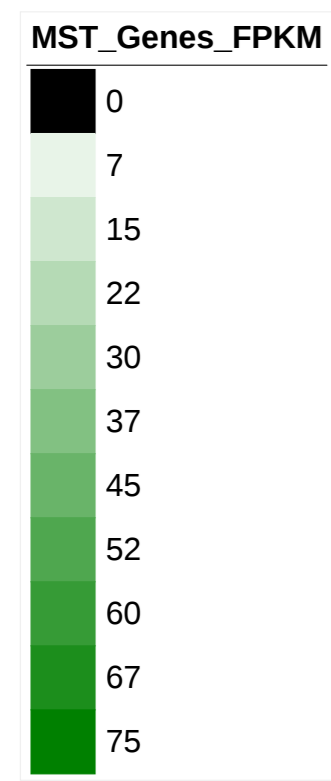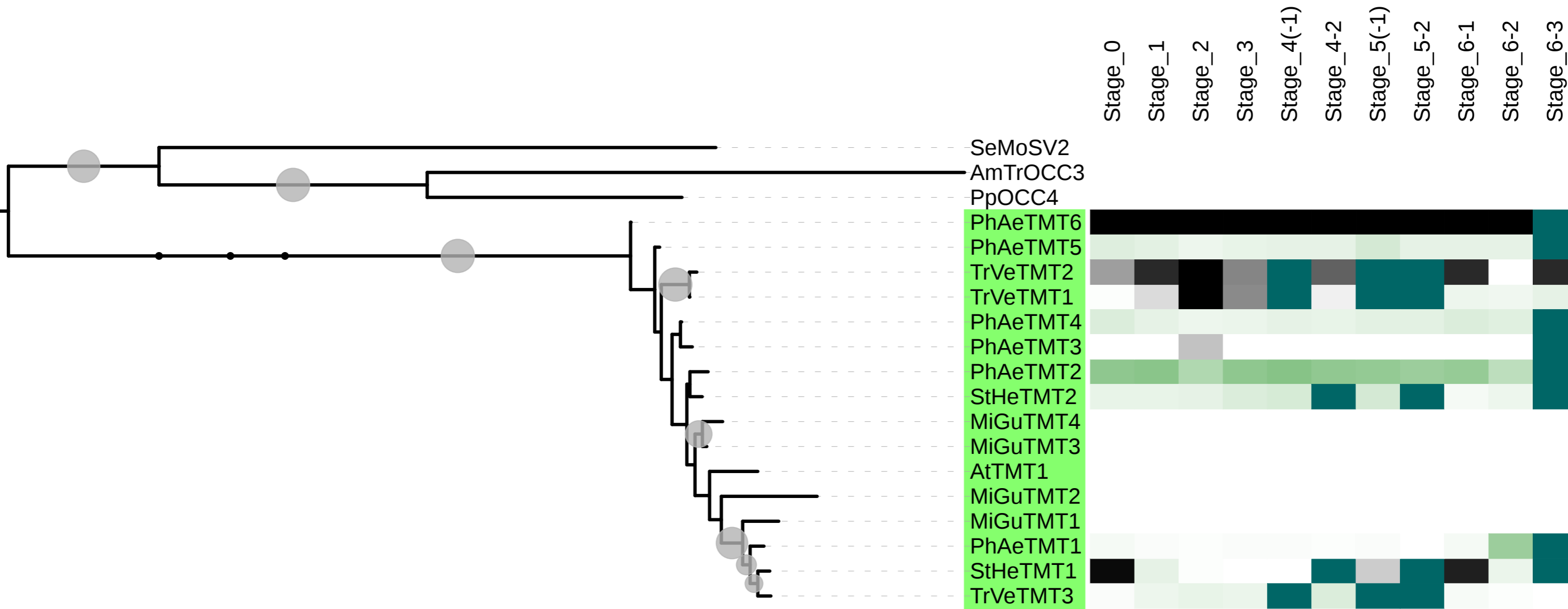

Supplement: Supplementary file 3 — The TMT clade of MSTs from parasitic plant transcriptomes and non-parasitic Mimulus genome. Note the slight increase in TMT expression during pre-connection and haustorial connection phases. (PDF 24 kb) [file 12870_2019_1786_MOESM3_ESM.pdf]

Tree scale: 0.1

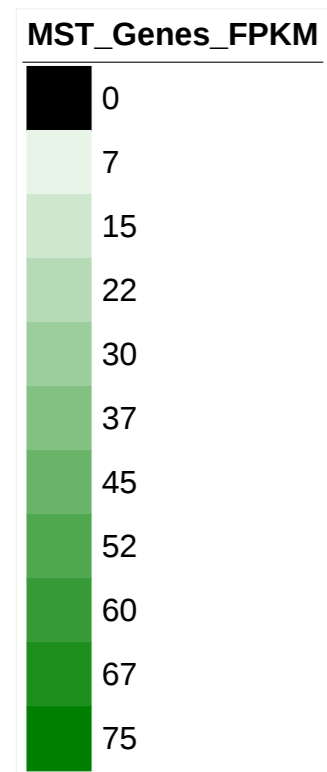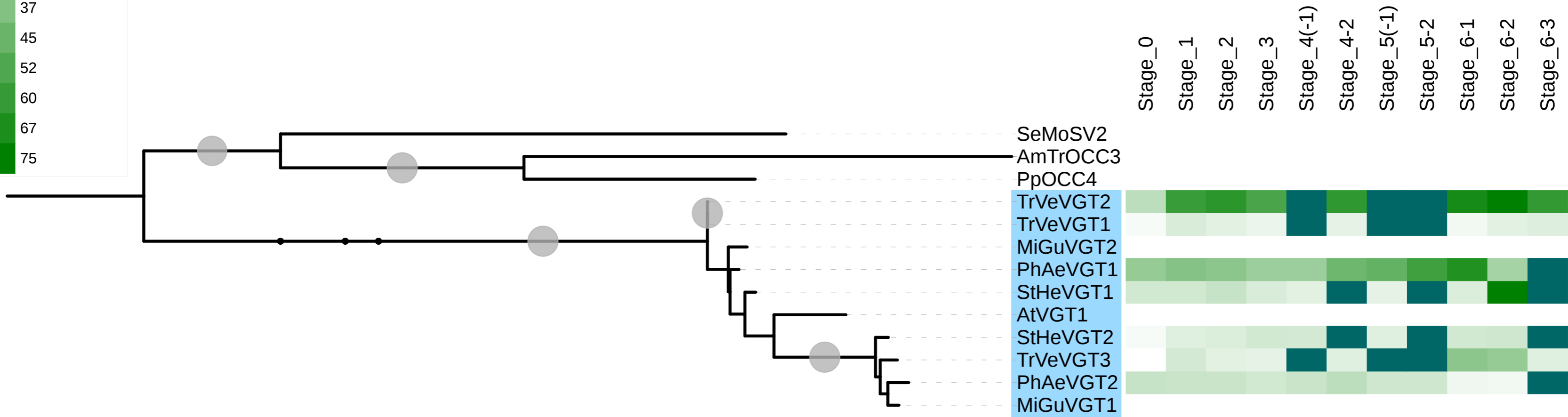

Supplement: Supplementary file 4 — The VGT clade of MSTs from parasitic plant transcriptomes and non-parasitic Mimulus genome. Note the tendency to express more strongly in stages 6–1 and 6–2. (PDF 23 kb) [file 12870_2019_1786_MOESM4_ESM.pdf]

MST Genes FPKM

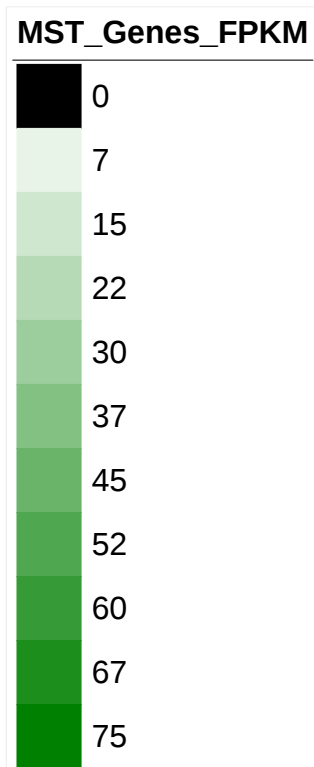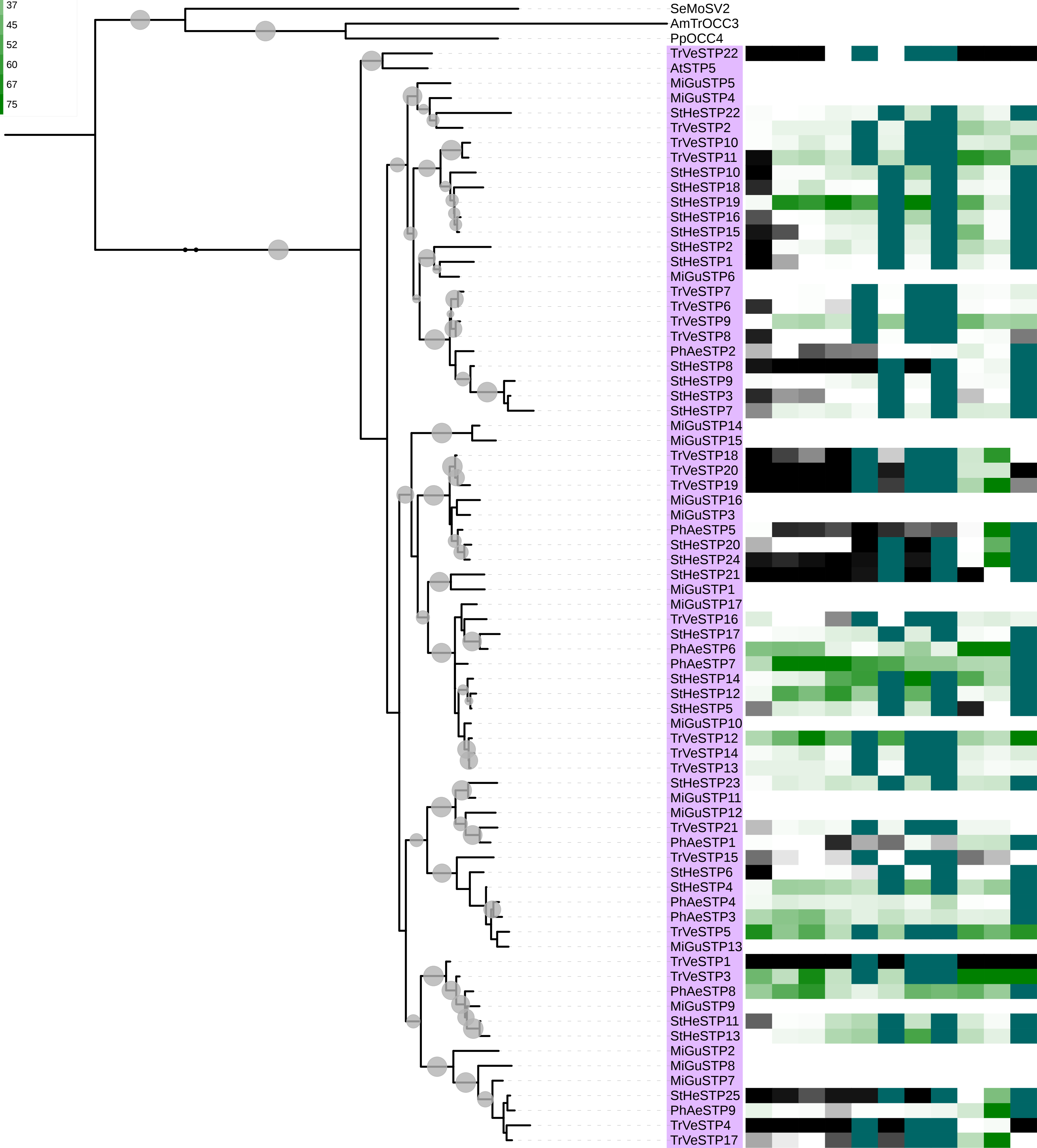

Supplement: Supplementary file 5 — The STP clade of MSTs from parasitic plant transcriptomes and non-parasitic Mimulus genome. Note the increase in expression during post-attachment stages in Triphysaria. (PDF 44 kb) [file 12870_2019_1786_MOESM5_ESM.pdf]

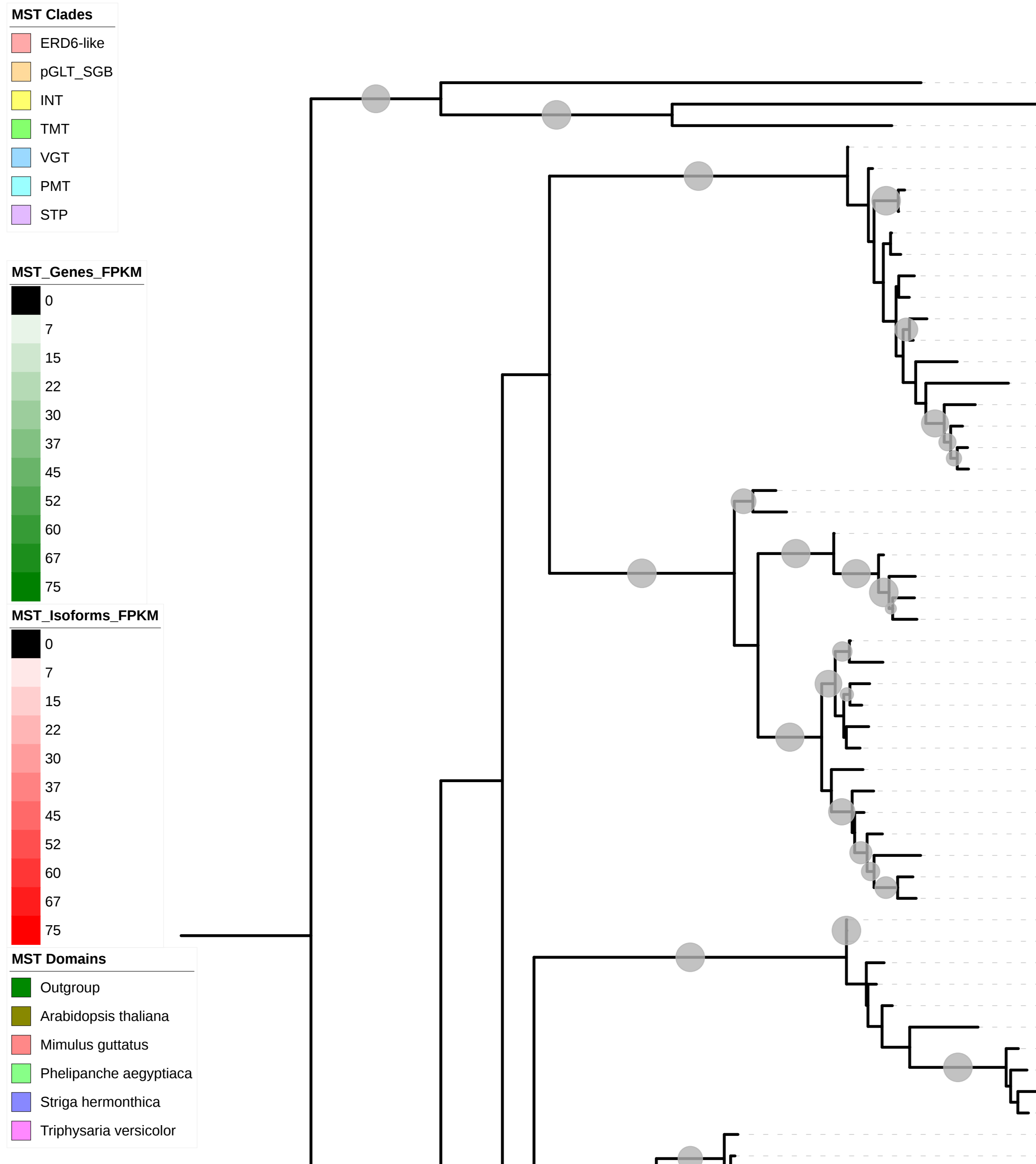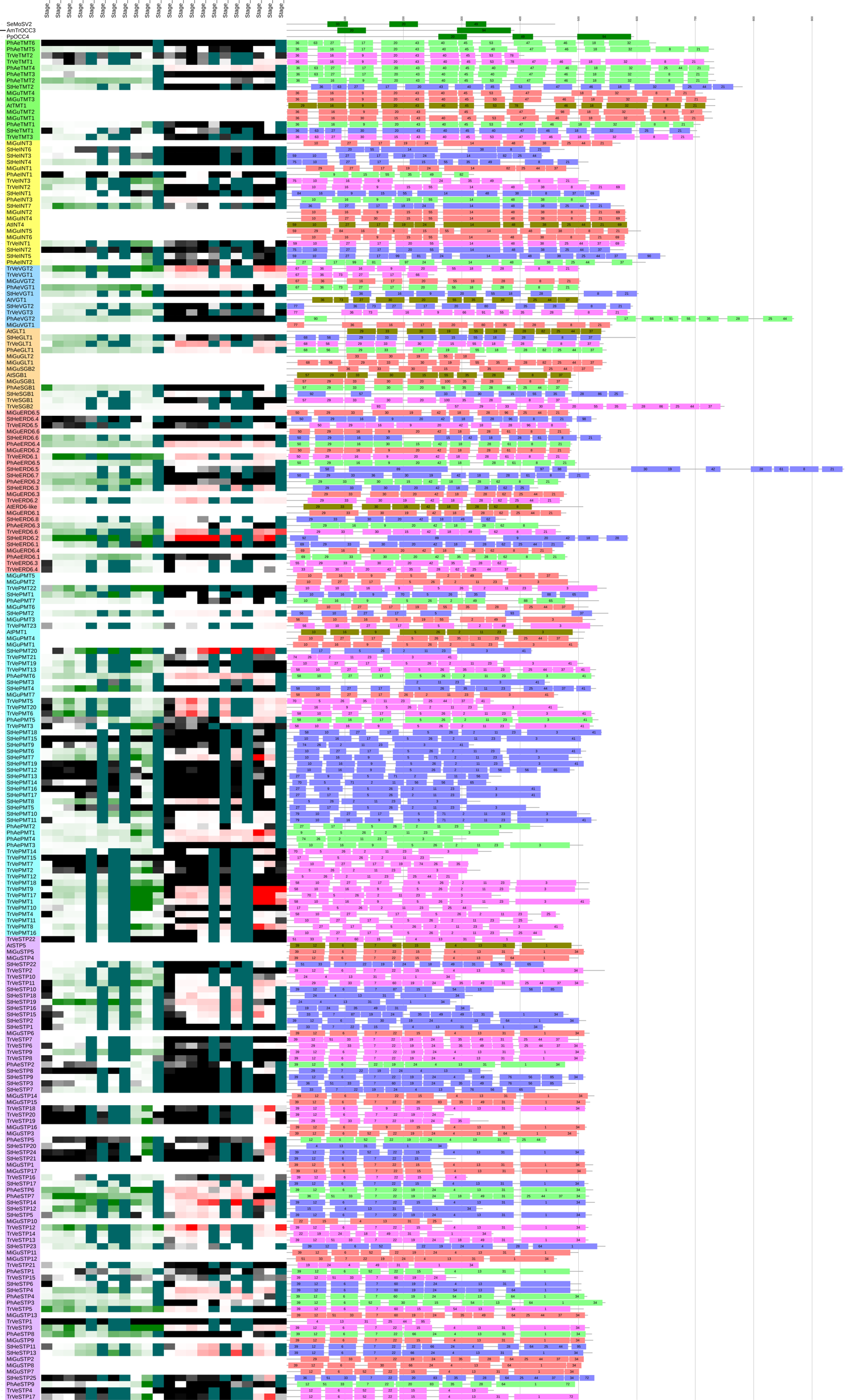

Supplement: Supplementary file 7 — Maximum likelihood tree of the MST genes, with heat map and domain architectures. Note the general increase in expression with each successive stage of the life cycle. (PDF 158 kb) [file 12870_2019_1786_MOESM7_ESM.pdf]

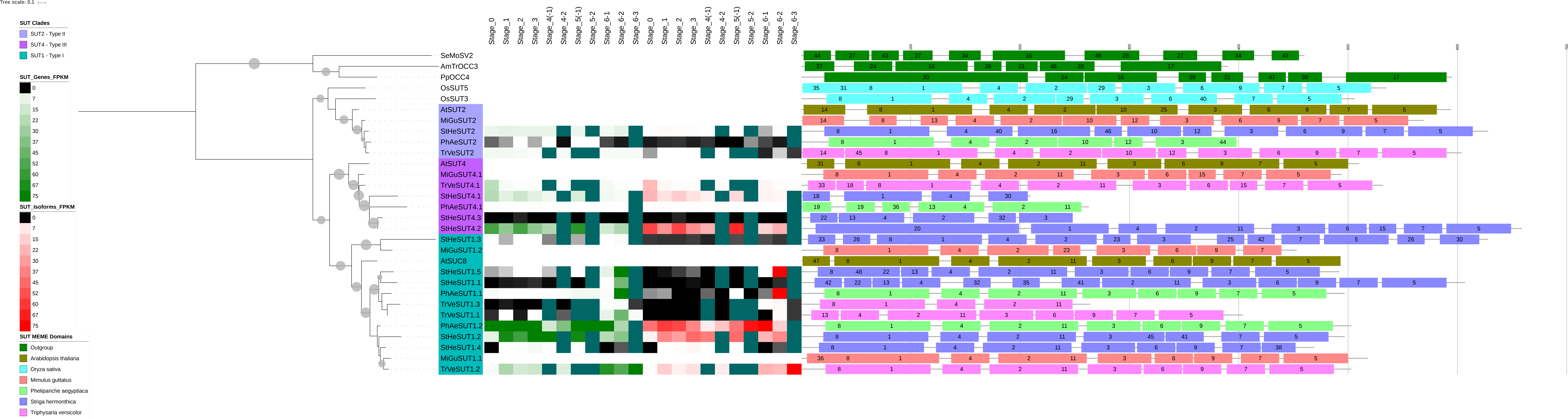

Supplement: Supplementary file 8 — Maximum likelihood tree of the SUT genes, with heat map and domain architectures. Note that in the SUT1 clade, there is a tendency for genes to express more strongly in later life cycle stages, culminating in a sharp increase in expression in stage 6–2. (PDF 43 kb) [file 12870_2019_1786_MOESM8_ESM.pdf]
