# Supplementary material for: Genome-wide identification of MST, SUT and SWEET family sugar transporters in root parasitic angiosperms and analysis of their expression during host parasitism
Source: BMC Plant Biol. 2019 May 14;19:196. doi: 10.1186/s12870-019-1786-y (PMC6515653; doi:10.1186/s12870-019-1786-y)
Supplement: Supplementary file 9 — Maximum likelihood tree of the SWEET genes, with heat map and domain architectures. Note the strong tendency to show higher levels of expression during the reproductive stage 6–2, with some exceptions in clades II and III. (PDF 73 kb) [file 12870_2019_1786_MOESM9_ESM.pdf]

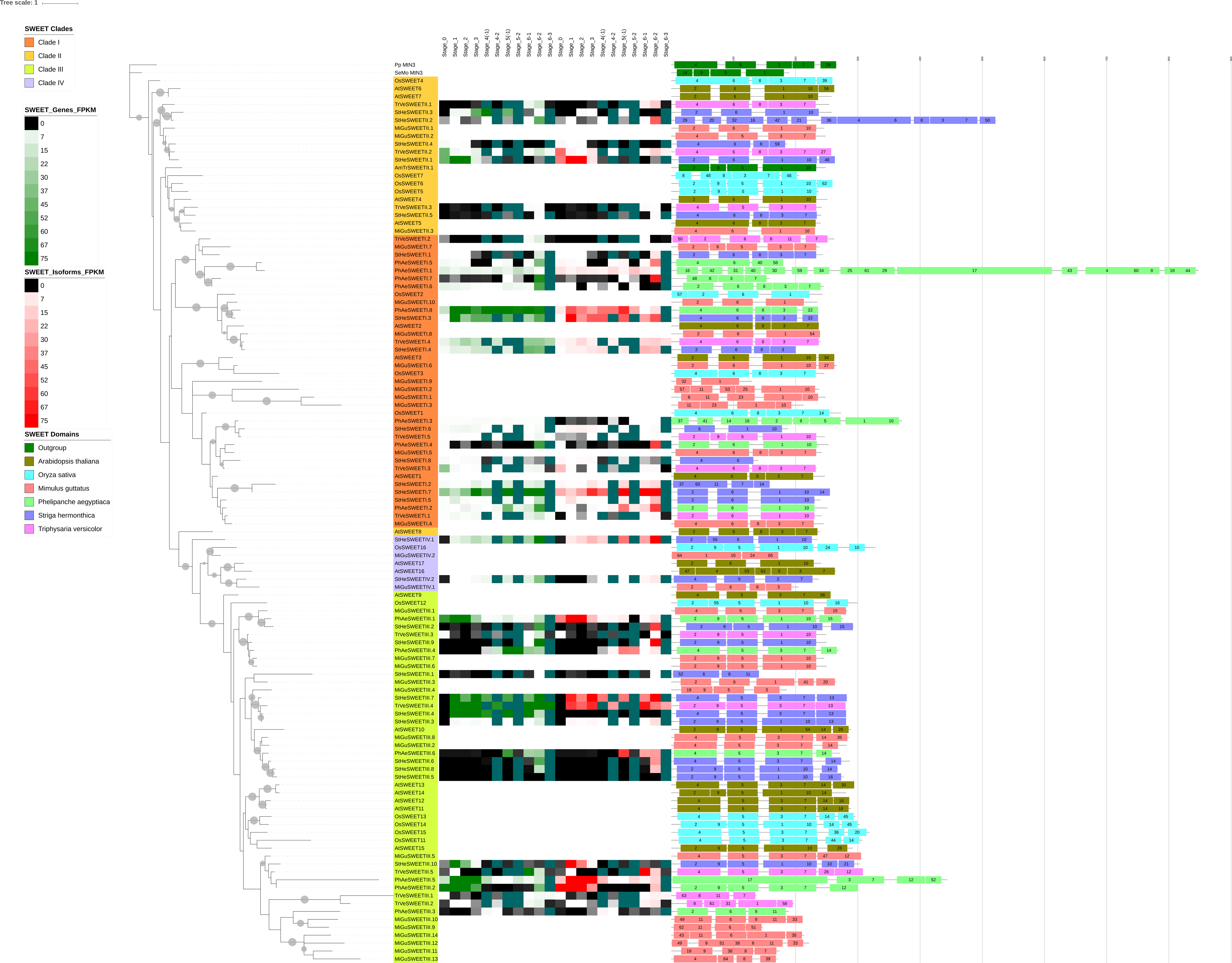
Tree scale: 1
SWEET Clades
Clade I
Clade II
Clade III
Clade IV
SWEET_Genes_FPKM
0
7
15
22
30
37
45
52
60
67
75
SWEET_Isoforms_FPKM
0
7
15
22
30
37
45
52
60
67
75
SWEET Domains
Outgroup
Arabidopsis thaliana
Oryza sativa
Mimulus guttatus
Phelipanche aegyptiaca
Striga hermonthica
Triphysaria versicolor
Stage_0
Stage_1
Stage_2
Stage_3
Stage_4(-1)
Stage_4-2
Stage_5(-1)
Stage_5-2
Stage_6-1
Stage_6-2
Stage_6-3
Stage_0
Stage_1
Stage_2
Stage_3
Stage_4(-1)
Stage_4-2
Stage_5(-1)
Stage_5-2
Stage_6-1
Stage_6-2
Stage_6-3
Pp MtN3
SeMo MtN3
OsSWEET4
AtSWEET6
AtSWEET7
TrVeSWEETII.1
StHeSWEETII.3
StHeSWEETII.2
MiGuSWEETII.1
MiGuSWEETII.2
StHeSWEETII.4
TrVeSWEETII.2
StHeSWEETII.1
AmTrSWEETII.1
OsSWEET7
OsSWEET6
OsSWEET5
AtSWEET4
TrVeSWEETII.3
StHeSWEETII.5
AtSWEET5
MiGuSWEETII.3
TrVeSWEETI.2
MiGuSWEETI.7
StHeSWEETI.1
PhAeSWEETI.5
PhAeSWEETI.1
PhAeSWEETI.7
PhAeSWEETI.6
OsSWEET2
MiGuSWEETI.10
PhAeSWEETI.8
StHeSWEETI.3
AtSWEET2
MiGuSWEETI.8
TrVeSWEETI.4
StHeSWEETI.4
AtSWEET3
MiGuSWEETI.6
OsSWEET3
MiGuSWEETI.9
MiGuSWEETI.2
MiGuSWEETI.1
MiGuSWEETI.3
OsSWEET1
PhAeSWEETI.3
StHeSWEETI.6
TrVeSWEETI.5
PhAeSWEETI.4
MiGuSWEETI.5
StHeSWEETI.8
TrVeSWEETI.3
AtSWEET1
StHeSWEETI.2
StHeSWEETI.7
StHeSWEETI.5
PhAeSWEETI.2
TrVeSWEETI.1
MiGuSWEETI.4
AtSWEET8
StHeSWEETIV.1
OsSWEET16
MiGuSWEETIV.2
AtSWEET17
AtSWEET16
StHeSWEETIV.2
MiGuSWEETIV.1
AtSWEET9
OsSWEET12
MiGuSWEETIII.1
PhAeSWEETIII.1
StHeSWEETIII.2
TrVeSWEETIII.3
StHeSWEETIII.9
PhAeSWEETIII.4
MiGuSWEETIII.7
MiGuSWEETIII.6
StHeSWEETIII.1
MiGuSWEETIII.3
MiGuSWEETIII.4
StHeSWEETIII.7
TrVeSWEETIII.4
StHeSWEETIII.4
StHeSWEETIII.3
AtSWEET10
MiGuSWEETIII.8
MiGuSWEETIII.2
PhAeSWEETIII.6
StHeSWEETIII.6
StHeSWEETIII.8
StHeSWEETIII.5
AtSWEET13
AtSWEET14
AtSWEET12
AtSWEET11
OsSWEET13
OsSWEET14
OsSWEET15
OsSWEET11
AtSWEET15
MiGuSWEETIII.5
StHeSWEETIII.10
TrVeSWEETIII.5
PhAeSWEETIII.5
PhAeSWEETIII.2
TrVeSWEETIII.1
TrVeSWEETIII.2
PhAeSWEETIII.3
MiGuSWEETIII.10
MiGuSWEETIII.9
MiGuSWEETIII.14
MiGuSWEETIII.12
MiGuSWEETIII.11
MiGuSWEETIII.13
